# Practical dietary interventions to prevent cardiovascular disease suitable for implementation in primary care: an ADAPTE-guided systematic review of international clinical guidelines

**DOI:** 10.1186/s12966-023-01463-9

**Published:** 2023-07-28

**Authors:** Delphine Le Goff, Naomi Aerts, Michele Odorico, Morgane Guillou-Landreat, Gabriel Perraud, Hilde Bastiaens, Geofrey Musinguzi, Jean-Yves Le Reste, Marie Barais

**Affiliations:** 1 Department of General Practice, University of Western Brittany, 22, Av. Camille Desmoulins, Brest, 29238 France; 2 ER 7479 SPURBO, University of Western Brittany, 22, Av. Camille Desmoulins, Brest, 29238 France; 3 Department of Primary and Interdisciplinary Care, Faculty of Medicine and Health Sciences, University of Antwerp, Antwerp, 2610 Belgium; 4 Global Health Institute, Faculty of Medicine and Health Sciences, University of Antwerp, Antwerp, 2610 Belgium; 5 Department of Disease Control and Environmental Health, School of Public Health, Makerere University, Kampala, Uganda

**Keywords:** Heart diseases, Primary prevention, Diet, Primary health care, Population health management

## Abstract

**Purpose:**

Cardiovascular diseases (CVD) are the leading cause of death globally. The current model of care for high-income countries involves preventive medication and highly trained healthcare professionals, which is expensive and not transposable to low-income countries. An innovative, effective approach adapted to limited human, technical, and financial resources is required. Measures to reduce CVD risk factors, including diet, are proven to be effective. The survey “Scaling-up Packages of Interventions for Cardiovascular disease prevention in selected sites in Europe and Sub-Saharan Africa” aims to develop non-pharmacological cardiovascular prevention and control programs in primary care and community settings in high, middle, and low-income countries. This review aims to identify the existing, validated dietary interventions for primary CVD prevention from national and international clinical guidelines that can be implemented in primary care and communities.

**Methods:**

A systematic review of CVD prevention guidelines was conducted between September 2017 and March 2023 using the Turning Research Into Practice medical database, the Guidelines International Network, and a purposive search. The ADAPTE procedure was followed. Two researchers independently conducted the searches and appraisals. Guidelines published after 01/01/2012 addressing non-pharmacological, dietary interventions for primary CVD prevention or CVD risk factor management, in the adult general population in primary care or in community settings were included and appraised using the Appraisal of Guidelines Research and Evaluation II score. Individual dietary recommendations and the studies supporting them were extracted. Then supporting data about each specific dietary intervention were extracted into a matrix.

**Results:**

In total, 1375 guidelines were identified, of which 39 were included. From these, 383 recommendations, covering 10 CVD prevention themes were identified. From these recommendations, 165 studies for effective dietary interventions for CVD prevention were found. Among these, the DASH diet was the most effective on multiple CVD risk factors. Combining diet with other interventions such as exercise and smoking cessation increased efficacy. No guidelines provided detailed implementation strategies.

**Conclusion:**

The DASH diet combined with other interventions was the most effective on an individual basis. However, expansion in the wider population seems difficult, without government support to implement regulations such as reducing salt content in processed food.

**Trial Registration:**

Clinical Trials NCT03886064

## Abstract (plain language summary)

Heart disease is the leading cause of death around the world. Strategies to prevent heart disease in high-income countries rely on medications and the skills of highly trained healthcare professionals. However, this is expensive and unsuitable for low-income countries. Consequently, an innovative, effective approach, which can be adapted to countries with limited human, technical and financial resources is needed. A program called SPICES was developed to identify strategies other than medication to prevent and control heart disease. This program reviewed the evidence for smoking cessation, physical activity, and dietary strategies, which may be useful to prevent heart disease in communities with limited resources.

In this review, the investigators searched online databases to find clinical guidelines that recommended dietary strategies to manage heart disease worldwide. The information found from this search revealed that the DASH diet, inspired by the Mediterranean diet, helps with weight loss, and improves blood pressure and cholesterol levels making it the most effective diet for preventing heart disease. It is even more effective if it is combined with other strategies such as exercise, stopping smoking or reducing the amount of alcohol consumed. However, this works well for individuals but is difficult to expand to the wider population. Therefore, government support is needed to implement regulations such as reducing salt content in processed food.

## Introduction

During the twenty-first Century, cardiovascular diseases (CVD) have become the leading global cause of death, resulting in 17.9 million deaths in 2019 [1]. In Europe, CVD costs are estimated at 169 billion Euros annually [2]. Furthermore, CVD death rates are higher in the lower socioeconomic levels between and within countries with three quarters of CVD deaths occurring in low-income countries [3].

The current model of care for high-income countries involves preventive medication and highly trained healthcare professionals but is becoming increasingly difficult to maintain due to high costs and is not transposable to low-income countries. An innovative, effective approach adapted to limited human, technical, and financial resources is required. Inspired by the progress made in HIV and AIDS treatment in Sub-Saharan Africa (SSA), the World Health Organization (WHO) created the ICCC Framework (Innovative Care for Chronic Conditions). This framework redirects the current health policies from being population-centered to patient-centered, involving patients, families, and communities [3]*.*

CVD occurrence increases predictably with accumulating CVD risk factors. Measures to reduce modifiable CVD risk factors, including diet, are proven to be effective. For example, in the general population, it is estimated that reducing cholesterol by 10% could decrease CVD mortality by 20% [4] and adopting a Mediterranean diet could reduce cardiovascular event occurrence by 30% [5]*.* Furthermore, CVD prevention interventions such as policies promoting healthy eating, smoking cessation, and physical activity, are cost-effective on international and national levels. It has been estimated that their costs would not exceed 4% of current health expenditure in high-income countries and 1–2% in low-income countries [6]. Also, behavioral changes are cost-effective on an individual level [7].

Consequently, the “Scaling-up Packages of Interventions for Cardiovascular disease prevention in selected sites in Europe and Sub-Saharan Africa” (SPICES) study was developed to evaluate and implement a comprehensive non-pharmacological cardiovascular prevention and control program in primary care and community settings in high, middle, and low-income countries [8]. The study involved systematically reviewing systematic clinical practice guidelines for smoking cessation, physical activities, and diet to identify best practice recommendations for reducing CVD. The smoking cessation and physical activity reviews have been previously published [9].

This review aims to identify the existing, validated dietary interventions for primary cardiovascular prevention from national and international clinical practice guidelines including European Union Countries, the UK, and Sub–Saharan Africa that can be implemented in primary care and communities.

## Material and methods

### Information sources and search

A systematic review of CVD prevention guidelines was conducted at the beginning of the SPICES study between September 2017 and January 2018 using the TRIP (Turning Research Into Practice) medical database and the International Guidelines Library of the Guidelines International Network (G-I-N). Subsequently, an update was performed in March 2023 prior to publication to ensure all recent guidelines were included. Following the ADAPTE procedure, the PIPOH tool (Population, Intervention, Professional/Patient, Outcome, Healthcare setting) was used to define the database search queries [10]. Population was defined as a primary care general population, free from cardiovascular disease. The Interventions were those focusing on cardiovascular risk factors including diabetes, hypertension, smoking, sedentary lifestyle, unhealthy diet, excess weight, or obesity. Professionals/Patients were any healthcare professional working in primary care or lay people. The Outcomes were reduced morbidity or mortality. The Healthcare setting and context was primary care. Search queries were “cardiovascular disease prevention” and “cardiovasc* prevention OR risk* OR risico* OR risque*”.

Then, a purposive search for every national clinical guideline used in the SPICES countries was performed. Guidelines included the Haute Autorité de Santé for France, the Tijdschrift Huisarts and the Nederland Huisartsen Genootschap for Belgium, the National Institute for Health and Care Excellence (NICE) for the United Kingdom, the European Society of Cardiology for European Countries for South Africa and the WHO for Uganda.

The review was reported following the 2020 PRISMA reporting guidelines.

### Eligibility criteria

Guidelines were included if they addressed non-pharmacological, dietary interventions for primary CVD prevention or CVD risk factor management, in the adult general population in primary care or in community settings. At least one patient outcome measure used for CVD risk assessment, such as mortality and morbidity, had to be reported in the guidelines. They had to be published after 01/01/2012 and be the latest version for revised guidelines. Guidelines could be written in English, French or Dutch.

Guidelines were excluded if they focused solely on specific populations such as elderly people, infants, children, pregnant women, or people with cancer. Guidelines focusing on secondary or tertiary prevention or only addressing cardiovascular risk assessment, pharmacological or surgical interventions, or specific conditions (such as type 1 diabetes, familial hypercholesterolemia) were excluded as were guidelines published before 2012 as these were considered out-of-date. Guidelines with no free full text availability were excluded as the consortium felt that recommendations to healthcare professionals or stakeholders should be freely accessible.

Searches were independently conducted by two researchers with a merging of results at each step of the review. Discrepancies were resolved by the two researchers and the study scientific committee.

### Guideline, recommendation, and study selection

Two data collection phases were performed. During the first phase, the two researchers independently selected the guidelines. In the second phase, guidelines were evaluated according to the “Appraisal of Guidelines Research and Evaluation II” (AGREE II) tool integrated in the ADAPTE procedure [10]. The AGREE II tool consists of 23 items arranged into six domains: scope and purpose, stakeholder involvement, rigor of development, clarity of presentation, applicability, and editorial independence. The two researchers independently scored each guideline by domain. An overall assessment was then performed and ranged from 1 (strongly disagree) to 7 (strongly agree). This score was independent from the other domain scores.

A scientific committee consisting of three members was created to select guidelines based on the overall assessment scores. If the overall assessment scores from both researchers were equal to or higher than 5 for a selected guideline, it was included in the review. The difference between the overall assessment scores could not exceed one. If the difference was greater than one, an overall assessment score was found by consensus between the two researchers and the scientific committee.

Once the guidelines were selected, they were inspected for specific recommendations for effective, dietary interventions for primary CVD prevention. Recommendations with an A or B level of evidence, or Class I or Strong and/or 1 +  + ,1 + , 2 +  + , 2 + for NICE grading (regardless of level of evidence) were included. If an effective dietary intervention for primary CVD prevention with a pragmatic implementation strategy was supported by a study, this study, regardless of date, was considered for inclusion in a final matrix. Study exclusion criteria included study population under 50, gender specific study, and the absence of a control group for individual interventions.

**Fig. 1:**
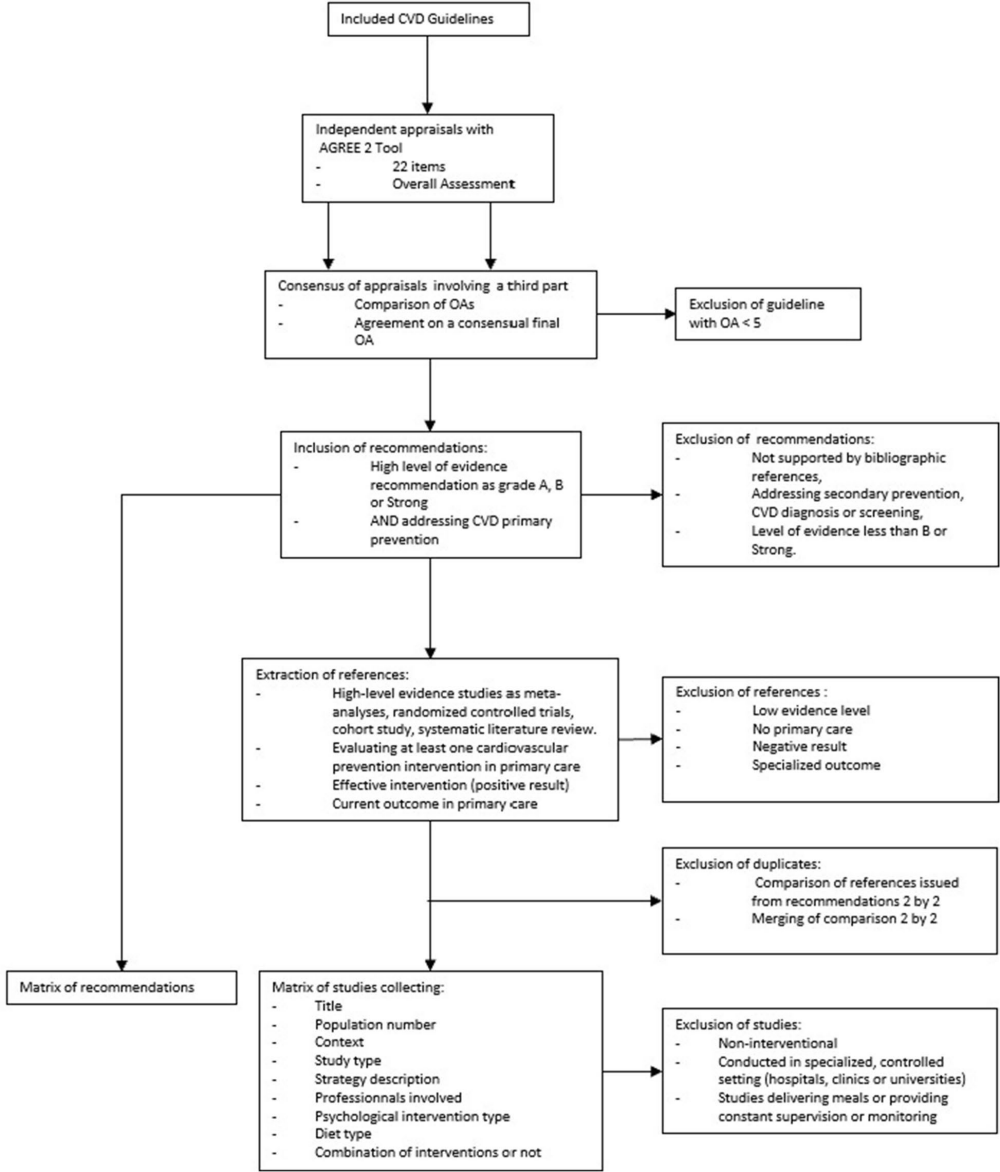
Selection process of guidelines, recommendations, and studies

As the recommendations in the different guidelines frequently cited the same studies, a duplicate elimination process was performed. The complete selection process of guidelines, recommendations and studies is described in Fig. 1.

### Data extraction and synthesis

For each included guideline, publication year or year of latest update, country, developing organization, language and title were extracted. From each included guideline a list of all dietary recommendations was created to provide an overview of relevant content. Recommendation themes were created inductively by grouping similar topic recommendations. The strength of recommendation, level of evidence, intervention description, outcomes, implementation strategies and evidence gaps were extracted from each recommendation if reported. The recommendations and studies they cited were then compiled into two matrices. After reading the full text, study title, implementation or intervention description, intervention frequency and duration, setting, material used, psychological model used where applicable, mass media use, and delivered intervention status were extracted into the matrices.

## Results

**Fig. 2:**
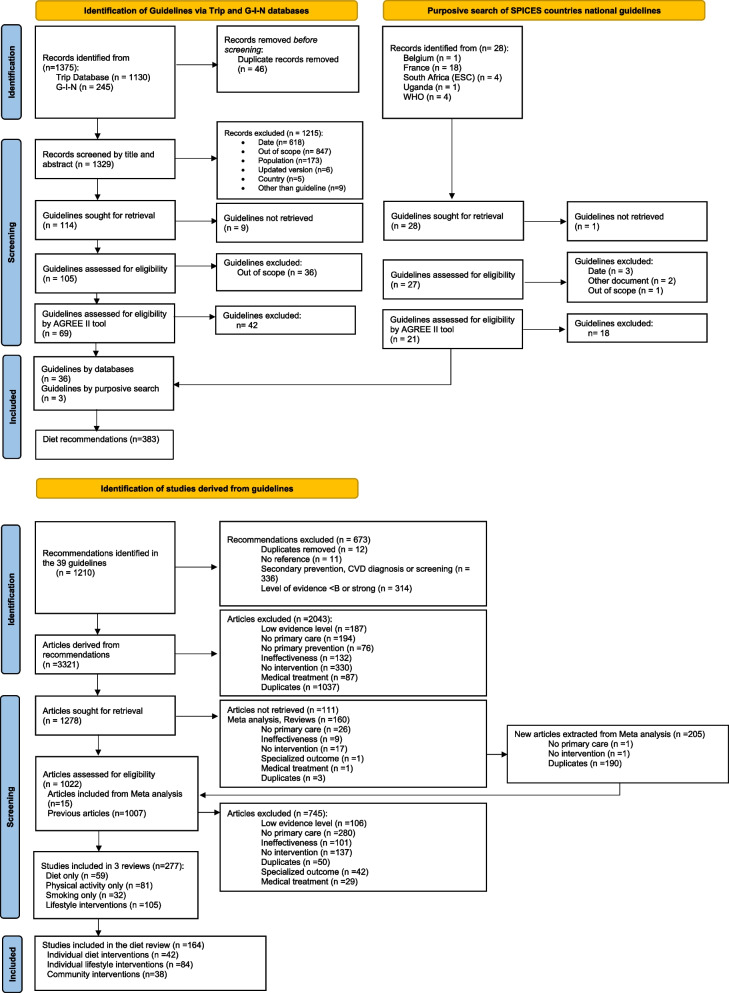
Selection of primary CVD prevention guidelines, recommendations and studies

A total of 39 primary CVD prevention guidelines were included (Fig. 2 and Table 1). Two guidelines were from the WHO and 37 were from high-income countries. None were by an organization from a middle- or low-income country.

**Table 1:** List of included guidelines addressing dietary interventions for primary CVD prevention

GUIDELINE	AUTHOR	COUNTRY	YEAR	RATING SYSTEM (RS)	LINK TO EVIDENCE
U.S. Preventive Services Task Force: Screening for and management of obesity in adults	USPSTF	USA	2012	USPSTF	Body of evidence after a table of graded recommendations
Canadian Diabetes Association 2013 Clinical practice guidelines for the prevention and management of diabetes in Canada	Canadian Diabetes Association	Canada	2013	GRADE	Major evidence cited in a specific paragraph before the recommendation box
2013 AHA/ACC guidelines on lifestyle management to reduce cardiovascular risk	ACC/AHA)	USA	2014	ACC/AHA Classification of Recommendation/Level of Evidence (COR/LOE) construct	Table to link the general critical question (CQ) + specific evidence statement developed in the CQ
2013 AHA/ACC/TOS guidelines for the management of overweight and obesity in adults	ACC / AHA / TOS	USA	2014	ACC/AHA (COR/LOE)	Table to link the general CQ + specific evidence statement developed in the CQ
Team-based care and improvement of blood pressure control: recommendation of the Community Preventive Services Task Force	Community Preventive Services Task Force	USA	2014	No RS	References at the end of the article (no link)
Clinical practice guidelines for the management of overweight and obesity in adults, adolescents, and children in Australia	NHMRC	Australia	2014	NHMRC	Body of evidence after a table of graded recommendations
Lipid modification: cardiovascular risk assessment and the modification of blood lipids for the primary and secondary prevention of cardiovascular disease. CG181	NICE	UK	2014	GRADE	Body of evidence first then recommendations
Behavior change: individual approaches (PH49)	NICE	UK	2014	No RS	Isolated body of evidence (specific chapter)
Cardiovascular disease prevention (PH25)	NICE	UK	2014	No RS	Isolated body of evidence (specific chapter)
Guidelines for the management of absolute cardiovascular disease risk	NVDPA	Australia	2014	NHMRC	Body of evidence after a table of graded recommendations
Prevention and Control of Noncommunicable Diseases. Guidelines for primary health care in low-resource settings	WHO	World	2015	GRADE	Body of evidence after each recommendation
Maintaining a healthy weight and preventing excess weight gain among adults and children	NICE	UK	2015	No RS	Isolated body of evidence (specific chapter)
Recommendations for prevention of weight gain and use of behavioral and pharmacological interventions to manage overweight and obesity in adults in primary care	Canadian Task Force on Preventive Health Care	Canada	2015	GRADE	Major evidence cited in a specific paragraph before the recommendation box
Sugars intake for adults and children	WHO	World	2015	GRADE	Body of evidence after each recommendation
Hypertension evidence-based nutrition practice guideline	ADA	USA	2016	Academy’s nutrition care process RS	Selected body of evidence after each recommendation
Guideline for the management of dyslipidemia for the prevention of cardiovascular disease in the adult	Canadian 2016 Lipids Panel Members	Canada	2016	GRADE	Body of evidence after each recommendation
Recommended Dietary Pattern to Achieve Adherence to the American Heart Association/American College of Cardiology (AHA/ACC) Guidelines: A Scientific Statement from the American Heart Association	ACC/AHA	USA	2016	ACC/AHA (COR/LOE)	Main references throughout the text
The 2015 Dutch food-based dietary guidelines	Committee Dutch Dietary Guidelines	Netherlands	2016	No RS	Main references throughout the text
Risk estimation and the prevention of cardiovascular disease	SIGN	UK	2017	SIGN	Body of evidence first then recommendations
AACE/ACE Guidelines for the management of dyslipidemia and prevention of cardiovascular disease	AACE/ACE	USA	2017	AACE/ACE	Isolated body of evidence (specific chapter)
Hypertension in adults: diagnosis and management (NG 136)	NICE	UK	2019	No RS	Isolated body of evidence (specific chapter)
ACC/AHA Guideline on the primary prevention of cardiovascular disease	ACC/AHA	USA	2019	ACC/AHA (COR/LOE)	Body of evidence after each recommendation
Updated Cardiovascular prevention guideline of the Brazilian society of cardiology	Brazilian Society of Cardiology	Brazil	2019	Brazilian Society of Cardiology RS	Main references throughout the text
Primary Prevention of ASCVD and T2DM in Patients at Metabolic Risk: An Endocrine Society Clinical Practice Guideline	ADA/ European Endocrine Society	USA/Europe	2019	GRADE	Body of evidence after a table of graded recommendations
Innovation to Create a Healthy and Sustainable Food System: A Science Advisory from the American Heart Association	AHA	USA	2019	No RS	Main references throughout the text
ESC/EAS Guidelines for the management of dyslipidaemias: lipid modification to reduce cardiovascular risk	ESC/EAS	Europe	2020	ESC RS	Main references throughout the text
Atherosclerotic cardiovascular disease risk assessment & management	Qatari Ministry of Health	Qatar	2020	Qatari RS	Main references throughout the text
VA/DoD clinical practice guideline for screening and management of overweight and obesity	VA/DoD	USA	2020	GRADE	Body of evidence after each recommendation
VA/DoD clinical practice guidelines for the diagnosis and management of hypertension in the primary care setting	VA/DoD	USA	2020	GRADE	Body of evidence after each recommendation
VA/DoD clinical practice guidelines for the management of dyslipidemia for cardiovascular risk reduction	VA/DoD	USA	2020	GRADE	Body of evidence after each recommendation
Outil d'aide au repérage précoce et intervention brève: alcool, cannabis, tabac chez l'adulte	HAS	France	2021	No RS	Main references throughout the text
2021 Dietary Guidance to Improve Cardiovascular Health: A Scientific Statement From the American Heart Association	USPSTF	USA	2021	USPSTF RS	Report of the systematic review dedicated to one recommendation
2021 ESC Guidelines on cardiovascular disease prevention in clinical practice	ESC	Europe	2021	ESC RS	Main references throughout the text
Behavioral Counseling Interventions to Promote a Healthy Diet and Physical Activity for Cardiovascular Disease Prevention in Adults Without Cardiovascular Disease Risk Factors: US Preventive Services Task Force Recommendation Statement	USPSTF	USA	2022	USPSTF	Body of evidence after a table of graded recommendations
Obesity prevention (CG189)	NICE	UK	2022	No RS	Isolated body of evidence (specific chapter)
Comprehensive Management of Cardiovascular Risk Factors for Adults with Type 2 Diabetes: A Scientific Statement From the American Heart Association	AHA	USA	2022	No RS	Main references throughout the text
Standards of Care in Diabetes—2023	ADA	USA	2022	ADA RS	Body of evidence after a table of graded recommendations
Vitamin, Mineral, and Multivitamin Supplementation to Prevent Cardiovascular Disease and Cancer: US Preventive Services Task Force Recommendation Statement	USPSTF	USA	2022	USPSTF RS	Report of the systematic review dedicated to one recommendation
Preventing type 2 diabetes—population and community interventions (PH35)	NICE	UK	2022	No RS	Isolated body of evidence (specific chapter)

ADA- Academy of Nutrition and Dietetics, ACC/AHA- American College of Cardiology / American Heart Association, NHMRC-National Health and Medical Research Council, NICE- National Institute for Health and Care Excellence, NVDPA- National Vascular Disease Prevention Alliance, SIGN- Scottish Intercollegiate Guideline Network, TOS- The Obesity Society, USPSTF- U.S. Preventive Services Task Force, VA/DoD- Department of Veterans Affairs and the Department of Defense, WHO- World Health Organization,

From the selected guidelines, 383 dietary recommendations were extracted. Recommendation ratings indicate comparability and quality. The Canadian Diabetes Association, Canadian Task Force on Preventive Health Care, National Institute for Health and Care Excellence (NICE), Department of Veterans Affairs and the Department of Defense (VA/DoD), World Health Organization (WHO) and the collaboration between the American Diabetes Association (ADA) and the European Endocrine Society used the independent GRADE rating system which is a current gold standard [11]. The other organizations used their own rating systems or other organization’s rating system. However, 11guidelines had no recommendation rating system, including six NICE guidelines. The body of evidence could be linked to the recommendations in 29 guidelines and could not in the other 10 (Table 1).

Among the 383 dietary recommendations extracted, ten major themes were identified (Table 2 and Table 3). Of these, only 17 recommendations recommended against current strategies (4.4%). Recommendations addressing soya, nuts, stanol esters and sterols were contradictory, with the same recommendation strength. Vitamin D, potassium, calcium, magnesium, vitamins A, B, C, E, and omega-3 fatty acids supplements were also controversial. For example, the Academy of Nutrition and Dietetics promoted magnesium, calcium, potassium, and vitamin D supplementation while the European Society of Cardiology (ESC) and the Scottish Intercollegiate Guideline Network (SIGN) stated that the evidence did not support supplementation [12–14]. Only one guideline recommended that no alcohol should be consumed when 13 recommended only consuming small amounts [15].

**Table 2:** Summary of diet and lifestyle recommendations

	Healthy diet and macronutrients (*n* = 133)	Micronutrients and supplements (*n* = 20)	Weight management (*n* = 43)	Alcohol reduction (*n* = 14)	Lifestyle interventions (*n* = 54)
Recommendations for	Healthy diet characteristics by macronutrients: • Percentage of macronutrients (*n* = 2), • Increase in dietary fiber (*n* = 3) • Ideal macronutrients distribution (*n* = 2) • Low index carbohydrate diet (*n* = 3) • Polyunsaturated fatty acids and monounsaturated fatty acids (*n* = 2), • Reduce amounts of dietary cholesterol (*n* = 4) • Reduce calories from saturated fat (*n* = 11) • Reduce calories from trans-fat (*n* = 7)	Reduce sodium intake (*n* = 8)	Maintain weight loss of 3 to 5% at least (*n* = 11)	Consume small amounts of alcohol (*n* = 13)	Integrate physical activity (*n* = 9)
	Adopt Mediterranean dietary pattern (alternatively Nordic style diet (*n* = 2), vegetarian style diet) (*n* = 6)	Consume adequate amounts of dietary potassium (*n* = 2)	Diets for weight loss (*n* = 8)		Combine diet and physical activity (*n *= 17)
	Adopt DASH (*n* = 5)	Consume adequate amounts of dietary calcium and eventually adopt supplementation (*n* = 1)	Promote a healthy weight (*n* = 5)		Promote smoking cessation (*n* = 9)
	Variety of diets (*n* = 1)	Consume adequate amounts of dietary magnesium and eventually adopt supplementation (*n* = 1)	Promote weight loss (*n* = 10)		Develop multimodal interventions (n = 15)
	Healthy diet characteristics by type of food: •Fruits and vegetables (*n* = 12) •Grains and whole grains food (*n* = 12) •Legumes (*n* = 5) •Dairy products (*n* = 4) •Filtered coffee and tea (*n* = 3) •Nuts (*n* = 12) •Non tropical vegetable oils (*n* = 4) •Plant stanols or sterols (*n* = 1) •Consumption of fresh unprocessed food (n = 3) •Restrict refined carbohydrates and sweetened beverage (n = 11) •Reduce red meat and prefer fish, lean meats (*n* = 11)		Use weight loss maintenance programs (*n* = 5)		Integrate psychosociological factors to CVD prevention (n = 4)
	Use yeast rice nutraceuticals (*n* = 2)		Provide meal replacement for weight loss (*n* = 1)		
	Use functional food enriched with phytosterols (*n* = 2)				
	Space carbohydrates between meals for glyceamic control of diabetics (*n* = 1)				
Recommendations against	No evidence to support a soya intake recommendation (*n* = 2)	No evidence to recommend consumption of vitamine D to improve blood pressure (*n *= 1)	Do not use routinely very-low calorie diets (*n* = 2)	Do not drink alcohol (*n* = 1)	
	No evidence to support nuts intake recommendation (*n* = 1)	No evidence to recommend whether reducing sodium intake plus changing dietary intake of potassium, calcium, or magnesium on blood pression control (*n* = 1)	Do not use unduly restrictive and nutritionally unbalanced diets (*n* = 1)		
	No evidence of safety of consumption of stanol esters and plant sterols on the long term (*n* = 1)	No evidence to recommend supplementation of Vitamins A,B,C, E (*n* = 4)

**Table 3:** Summary of diet and lifestyle implementation recommendations

	Professionnals involved (*n* = 9)	Customization of intervention for individuals (*n* = 63)	Healthy family behaviours (*n* = 3)	Theoretical models (*n* = 25)	Public policies (*n* = 19)
Recommendations for	Type of professionnals involved (*n* = 4)	Adapt diet adaptation to patient's preference and conditions (*n* = 8)	Encourage healthy family behaviours (*n* = 3)	Behavioral change models and their implementation (*n* = 19)	Develop legislative measures (*n* = 4)
	Involve professionnals with specific expertise in nutrition (*n* = 3)	Involve person's partner or spouse (*n* = 1)		Shared decision model (*n* = 2)	Use Labelling and information on food items (*n* = 2)
	Ensure the muldisciplinary team is regularly trained and competent (*n* = 2)	Use comprehensive lifestyle programs (*n* = 19)		Match behavioral change to individual needs (*n* = 2)	Use economic incentives (*n* = 1)
		Intensity of lifestyle program (*n* = 6)		Use brief advice for alcohol reduction (n = 2)	Develop school educational campaigns (*n* = 1)
		Use telehealth and electronic weight-loss programs (*n* = 10)			Develop workplace interventions (*n* = 1)
		Use commercial weight-loss programs (*n* = 2)			Regulate fast-foods in community settings (*n* = 2)
		Characteristics of weight loss maintenance programs (*n* = 5)			Develop policies adressed to alcohol (n = 2)
		Gradual physical activity and diet improvement (*n* = 2)			Involve food manufacturers (n = 2)
		Diabetes care organizational model (*n* = 2)			Adress financial issues for healty diet (*n* = 1)
		Individualize self-management (*n* = 3)			Implement behavioural change in nutritional strategies (*n* = 1)
		Use community setting programs (*n* = 2)			

Within the non-pharmacological recommendations, 1210 studies were identified, of which 164 were included in the diet study matrix (Table 4). Among these studies, 42 investigated dietary interventions targeting individuals, 84 investigated lifestyle interventions targeting individuals, and 38 involved communities in CVD prevention using dietary interventions. All but two of the 164 studies, used a CVD prevention surrogate endpoint [16, 17]. The mean publication dates were 1999 for dietary interventions, 2004 for lifestyle interventions and 1994 for studies involving communities. Only nine studies involving communities were randomized controlled trials. The selection criteria for this category was therefore lowered to cohort studies to include the other 29 studies.

**Table 4:** Matrix of dietary interventions

Date of publication	Name of Reference	POPULATION	CONTEXT	Description of Strategy	Outcome	Outcome	Professionnal	Intervention type	Diet type
1982	Beard TC, Gray WR, Cooke HM, Barge R. Randomised controlled trial of a no-added-sodium diet for mild hypertension. Lancet 1982;2(8296):455–8	90 patients with anti hypertensive medication	Community	A shopping guide and address list for obtaining unsalted foods, including unsalted wholemeal bread and cakes made with potassium baking-powder, and unsalted restaurant meals. For 4 weeks they attended weekly 2 h small-group discussions at which slides were shown, recipes exchanged. Patients received the Australian dietary guidelines to eat less fat and sugar, more cereals and breads (preferably whole meal), and more fruits and vegetables -but were asked not to diet for weight reduction during the trial period	blood pressure	After coming to a mean sodium excretion rate of 35 mmol/24 h, the diet group finished with a lower mean SBP and DBP than the control group, and on about half the medication	?	Educational	The natural sodium content of a balanced diet was described as rather generous but safe. Milk (Na 21–28 mmol/1) was rationed and edible seaweed (kelp) prohibited. Patients received the Australian dietary guidelines to eat less fat and sugar, more cereals and breads (preferably wholemeal), and more fruits and vegetables -but were asked not to diet for weight reduction during the trial period.Australian dietary guidelines: more vegetables and fruit, particularly green, orange and red vegetables, such as broccoli, carrots, capsicum and sweet potatoes, and leafy vegetables like spinach, and legumes/beans like lentils. Grain (cereal) foods, particularly wholegrain cereals like wholemeal breads, wholegrain/high breakfast cereals, oats, wholegrain rice and pasta. Reduced fat milk, yoghurt and cheese varieties (reduced fat milks are not suitable for children under the age of 2 years as a main milk drink). Lean meats and poultry, fish, eggs, nuts and seeds and legumes/beans (except many Australian men would benefit from eating less red meat). Water instead of soft drinks, cordials, energy drinks, sports drinks and sweetened fruit juices and/or alcoholic drinks Most Australians need to eat less: Meat pies, sausage rolls and fried hot chips, Potato crisps, savoury snacks, biscuits and crackers Processed meats like salami, bacon and sausages Cakes, muffins, sweet biscuits and muesli bars Confectionary (lollies) and chocolate Ice-cream and desserts, Cream and butter, Jam and honey, Soft drinks, cordial, energy drinks and sports drinks Wine, beer and spirits
1985	MacMahon SW, Macdonald GJ, Bernstein L, Andrews G, Blacket RB. Comparison of weight reduction with metoprolol in treatment of hypertension in young overweight patients. *Lancet.* 1985; 8840: 1233–1236	42 men and 14 women, aged 20–55 years; supine diastolic blood pressure (phase V) 90–109 mm Hg, BMI greater than 26; no antihypertensive treatment	Community	In the weight reduction group, patients received an individually tailored dietary programme aimed at reducing caloric intake by 1000 cal per day, with protein, fat, and carbohydrate providing 15, 30, and 55% of calories, respectively. 7-day food logs were recorded in week 4 of the baseline and week 16 of the follow-up periods. Patients in the beta-blocker group were given metoprolol 100 mg twice a day. Patients in the placebo group were given one "metoprolol" placebo tablet twice a day. Subjects in all groups were seen individually every 3 weeks over 21 weeks (weeks 5 to 25)	blood pressure	Considering the weight-loss group, the fall in their systolic pressure of 13 mm Hg was significantly greater than that in the placebo group (7 mm Hg) but not different from that in the metoprolol group (10 mm Hg). Their fall in diastolic pressure (10 mm Hg) was greater than that in both the metoprolol (6 mm Hg) and placebo (3 mm Hg) groups	?	?	Weight loss diet type: an individually tailored dietary programme aimed at reducing caloric intake by 1000 cal per day, with protein, fat, and carbohydrate providing 15, 30, and 55% of calories, respectively
1985	Langford HG, Blaufox MD, Oberman A et al. Dietary therapy slows the return of hypertension after stopping prolonged medication. JAMA 1985;253 (5) 657- 664	584 patients	hypertensive population—primary prevention	The patients were seen at two-week intervals for 16 weeks and at monthly intervals thereafter, unless diastolic BP was 95 mmHg or higher, a development that required scheduling of weekly visits. Two types of dietary intervention groups were established: * The goal for one group was to decrease sodium intake to 70 mEq/day (4 g) and increase potassium intake to 100 mEq/day while maintaining body weight. * The goal for the second group was to reduce body weight Weight loss was achieved primarily by reducing caloric consumption, with relatively little emphasis on changing exercise. Nutritional intervention consisted of eight initial consecutive weekly group sessions, then monthly sessions thereafter plus individual consultation as needed	Decrease of hypertensive medication	At 56 weeks, randomization either to weight-loss group (mean loss of 4.5 kg [10 lb]) or to sodium-restriction group (mean reduction of 40 mEq/day) increased the likelihood of remaining without drug therapy, with an adjusted odds ratio of 2.17 for the sodium group and 3.43 for the weight group	?	decrease sodium intake OR reduce body weight by reducing calories	?
1986	Chalmers J, Morgan T, Doyle A. et al. Australian National Health and Medical Research Council dietary salt study in mild hypertension. J Hypertens 1986:4(suppl 6):S629-37	212 untreated subject with mild hypertension (DBP 90–100 mmHG)		Deit counseling, following 3 day of dietary intake record. 3 groups, no siginificant difference between them. Every 15 days, by dietitian and nurses. 12 weeks intervention.. Avoid adding salt. Eat extra fruits and vegetables. Drink fruit juices. Eat high potassium breakfast	blodd pressure	Two-hundred subjects completed the diet phase of 12 weeks. The falls in systolic and diastolic blood pressures (mmHg) in the diet phase were 7.7 ± 1.1 and 4.7 ± 0.7 (B), 8.9 ± 1.0 and 5.8 ± 0.6 (C) and 7.9 ± 0.9 and 4.2 ± 0.7 (D). These falls were all greater than those in the control group on an intention-to-treat analysis (P less than 0.005) but did not differ from each other	Dietician	Diet counseling 4 groups: - normal (control) - low sodium - low sodium /high potassium - high potassium	Diet counseling 4 groups: - normal (control) - low sodium - low sodium /high potassium - high potassium
1986	Baron JA, Schori A, Crow B, Carter R, Mann JI. A randomized controlled trial of low carbohydrate and low fat/high fiber diets for weight loss. Am J Public Health 1986; 76: 1293–1296	135 overweight subjects	One hundred thirty-five overweight subjects were recruited into the study with the help of six diet clubs and employee groups from the Oxford (United Kingdom) area	A commercial low-carbohydrate diet versus a commercial low-fat/high fiber diet. Spouses were randomized together. Each subject planned his/her own menus, with the assistance of the group leaders and the study investigators. Thus the dietary advice was in a form typical of that in currently used popular diets. Both diet regimens featured diet instruction sheets with the same format; therefore they were virtually identical in materials and procedures, although they differed substantially in content. At each center, participating subjects were given a general orientation to dieting. This included a brief discussion of behavioral techniques and the value of exercise, both of which were not specifically encouraged further. After instruction in the appropriate diet, each subject then participated in the normal operation of his or her group, which in all cases included weekly meetings. One of the study investigators visited each group regularly during the three-month diet period to offer encouragement and further instruction, if needed. Meanwhile, group leaders regularly weighed participants and avoided activities that might discourage or aid the weight loss of one study diet group compared to the other	weight loss	Dieters given low carbohydrate/low fiber dietary advice tended to lose more weight than those given a higher carbohydrate/higher fiber regimen (5.0 vs 3.7 kg on average at three months)	commercial diet programs leaders	commercial weekly group sessions	The two diet programs were both currently used commercially and had been designed for this popular use. Each focused on the restriction of one type of nutrient (carbohydrates or fat, depending on the diet). In each case, the diet was designed to be equivalent to 1,000–1,200 cal a day, although a few particularly active subjects were advised to liberalize the diet to 12 units (1,200- 1,400 cal) daily. The 10 carbohydrate units permitted a daily carbohydrate intake of at most 50 g (a less severe limitation than that imposed in low-carbohydrate ketogenic diets).'Similarly, the basic low fat diet restricted fat intake to at most 30 g a day. Each regimen had zero-unit foods that were not restricted directly. For the low-carbohydrate diet, these included meats and cheese; for the low-fat/high fiber diet, bread, potatoes, and fresh fruit. Increased intake of foods rich in fiber was specifically stressed to those in the low-fat group; this advice was not given to the low-carbohydrate dieters. Neither diet required a minimum of any food, although a half pint of milk per day was recommended to the low-carbohydrate dieters
1986	Croft PR, Brigg D, Smith S, Harrison CB, Branthwaite A, Collins MF. How useful is weight reduction in the management of hypertension? J R Coll Gen Pract. 1986; 36: 445–448	110 participants with hypertension	GP office	The patients attending the clinic were given active dietary advice for weight reduction by two dietitians working at the surgery. The groups were given identical advice, but the importance of weight reduction for blood pressure control was emphasized to the hypertensive patients. The hypertensive patients who did not attend the weight reduction clinic were seen only by the general practitioner. They were told that for six months their blood pressure would be checked periodically before any decision about specific treatment was taken. If patients in this group indicated that they intended to lose weight, they were not discouraged but were given no specific advice or diet sheets. Their plan to lose weight was recorded. All three groups of patients were given advice about modest restriction of salt use and reduction of excessive alcohol intake. No advice was given about smoking or exercise	weight loss	The weight loss achieved by obese hypertensive patients randomly allocated to a weight reduction programme in general practice was associated with significant reductions in both systolic and diastolic blood pressure when compared with a control group of obese hypertensive patients who were not dieting. The mean weight loss during the six months of the study was 6.5 kg for all the dieting hypertensive patients (a significant change) and 0.2 kg for the non-dieting hypertensive patients (a non-significant change)	2 dieticians	dietary advice	active dietary advice for weight reduction by two dietitians working at the surgery
1988	Wallace P, Cutler S, Haines A. Randomised controlled trial of general practitioner intervention in patients with excessive alcohol consumption. BMJ 1988; 297: 663–8	909 patients having drinking above the limits set for study and had not received advice	Medical Research Council's general practice research framework, mostly in rural or small urban settings	Patients randomised to the treatment group were contacted by their general practitioner and asked to attend for a brief interview. All general practitioners received a training session with use of a specially recorded video programme to illustrate the elements of the intervention. After an assessment interview about the pattern and amount of alcohol consumption and evidence of alcohol related problems and dependence (obtained by using the brief Edinburgh alcohol dependence scale2') patients were shown a histogram based on figures from a national survey of drinking habits to illustrate how their weekly consumption compared with that of the general population. Advice was given about the potential harmful effects of their current level of alcohol consumption, backed with the information booklet That's the Limit. Men were advised to drink not more than 18 U/week and women not more than 9 U/week. Where there was evidence of dependence on alcohol general practitioners were encouraged to advise abstinence. Patients were given a drinking diary, the front cover of which was a facsimile of an EClO prescription with the words "Cut Down on your Drinking!" The last page contained a guide to the alcohol content of a range of drinks. An initial follow up appointment one month later was offered to all patients; subsequent appointments at four, seven, and 10 months were at the discretion of the general practitioner. During these sessions the patient's drinking diary was reviewed and feedback given on the results of blood tests indicating evidence of damage due to alcohol	alcohol consumption	At one year a mean reduction in consumption of alcohol of 18.2 (SE 1.5) U/week had occurred in treated men compared with a reduction of 8.1 (1.6) U/week in controls (p less than 0.001) The proportion of men with excessive consumption at interview had dropped by 43.7% in the treatment group compared with 25.5% in controls (p less than 0.001). A mean reduction in weekly consumption of 11.5 (1.6) U occurred in treated women compared with 6.3 (2.0) U in controls (p less than 0.05), with proportionate reductions of excessive drinkers in treatment and control groups of 47.7% and 29.2% respectively	general practitioner (who had had a training session)	Brief intervention providing advice and information about how to reduce consumption + a drinking diary	no
1988	Weinberger MH, Cohen SJ, Miller JZ, Luft FC, Grim CE, Fineberg NS. Dietary sodium restriction as adjunctive treatment of hypertension. JAMA. 1988;259(17):2561–2565	114 hypertensive patients. All but 18 of these patients were receiving antihypertensive drugs and had achieved adequate blood pressure control	written or oral interventiion, veteran administration hypertension clinic	A food questionnaire designed to identify the individual's usual food preferences: the dietitians used the food preference questionnaires to review and identify sources of sodium in the usual diet and to provide alternatives that would permit reduction of dietary Dietary restriction lessons: held with both the patient and partner on three occasions Lessons 1 and 3 were conducted in the clinic and lesson 2 in the patient's home	blood pressure	Significant falls in blood pressure and body weight were observed with no significant correlations noted between the two variables, implying independence of these effects	Dieticians	Three lessons based on alternatives to usual diet to permit salt reduction	reduced sodium diet based on usual diet
1990	Singh RB, Rastogi SS, Mehta PJ, Mody R, Garg V. Effect of diet and weight reduction in hypertension.Nutrition. 1990; 6: 297–302	416 hypertensive participants	?	Weight reduction by a low-energy diet and a high-polyunsaturates-, fiber- and potassium-rich diet may be independently useful to hypertensives. Participants were randomized to either a low-energy cardiovasoprotective (CVP) diet, a low-energy usual diet, an optimal-energy CVP diet, or an optimal-energy, usual pre-experimental diet plus drug therapy in a single-blind and controlled fashion. Groups A and B received significant fewer calories per day than Groups C and D. Groups A and C also received significantly more calories per amount of complex carbohydrates, polyunsaturates, potassium, and magnesium than did Groups B and D. Dietary compliance and drug intake was checked weekly	Lipids	After 3 months, there was a significant fall in mean serum cholesterol (p less than 0.01) and mean serum triglycerides (p less than 0.05) in Group A compared with Group D. Group A and B patients had a loss of around 10 kg of mean body weight, with no weight change	?	? Dietary compliance and drug intake was checked weekly	a low-energy cardiovasoprotective (CVP) diet (Group A; n = 106), a low-energy usual diet (Group B; n = 104), an optimal-energy CVP diet (Group C; n = 104), or an optimal-energy, usual pre-experimental diet (Group D; n = 102) plus drug therap
1990	Campbell LV, Barth R, Gosper JK, Jupp JJ, Simons LA, Chisholm DJ. Impact of intensive educational approach to dietary change in NIDDM. Diabetes Care 1990;13(8):841–7	70 participants with non-insulin-dependent diabetes mellitus, suboptimal recent glycemic control, dietary fat intake ≥ 35% of total energy intake, and BMI ≥ 25 kg/m2, age of onset of diabetes > 30 yr, duration of diabetes > 3 mo, duration of current treatment > 1 mo, no education program attendance within the previous 6 mo,and English speaking,	primary care	Diet education Multiprofessionnal behavioral individual,dietary compliance, dietary intake, total cholesterol level, glycemic control Dietary instruction was greatly simplified. Subjects were told to decrease all fats and to increase legumes. Calories restriction and carbohydrate portions (exchanges) were not discussed, and types of fat were not distinguished. INTERVENTION• dietary (13.5 h in length) • conducted over 11 wk (total 22 h). The educational approach was based on a practical application of the cognitive motivational theory by Heckhausen and Kuhl o First: establish adequate reasons for behavioral change, the physician describes the major diabetic complications, and each subject visualizes the individual adverse effects of such afflictions (variable, goal value). o help the patient through the steps that lead to action. the physician encourages good metabolic control via healthy diet to help prevent complications. The simplified diet is then demonstrated by the dietitian, and patients are encouraged to practice devising realistic diet plans, i.e., plans they feel able to achieve (variable, expectancy). o Explore the many favorable and unfavorable individual consequences of making dietary changes, losing weight, and reducing the risk of complications with the psychologist, e.g., social inconvenience, better appearance, added expense, flatulence, and improved health (variable, instrumentality). o Patients then declare in writing whether they intend to follow the diet. o If this is the case, they are assisted to set realistic and specific goals for individual changes and to develop coping strategies for forseeable crisis situations	dietary compliance, dietary intake	The intensive approach was associated with significantly greater improvements in dietary compliance, dietary intake (complex carbohydrate, [P = 0.013], legumes [P less than 0.0001], fiber [P less than 0.0001], total fat [P less than 0.004], saturated fat [P less than 0.004]), and total cholesterol level (P = 0.007)	Multiprofessionnal team	cognitive motivational theory	simplified med diet, Subjects were told to decrease all fats and to increase legumes. Calories restriction and carbohydrate portions (exchanges) were not discussed, and types of fat were not distinguished
1992	Sciarrone SEG, Beilin LJ, Rouse IL, Rogers PB. A factorial study of salt restriction and a low-fat/high-fibre diet in hypertensive subjects. J Hypertens 1992;10:287–98	95 hypertensive subjects, mean age 53.5 years, consuming less than 30 ml ethanol/day were selected from community volunteers	community volunteers	Subjects followed either a low-sodium, low-fat/high-fibre diet (less than 60 mmol sodium/day; 30% fat energy; P:S ratio = 1; 30–50 g fibre/day) or a low-sodium, normal-fat/normal-fibre diet (less than 60 mmol sodium/day; 40% fat energy; P:S ratio = 0.3; 15 g fibre/day) for 8 weeks. Half of each group received 100 mmol/day NaCl and the remainder received placebo. Instructions:—no add salt to cooking, abstain from commercially prepared food, purchase "no salt variety " if available- low or no salt bread, butter and margarine,—avoid cheese, commercial cake, biscuits, chocolate- Avoid dining out. Provided with low sodium bread and no added salt butter. Low fat:—avoid frying- encouraged to consume 3 serving /day of vegetables 2/day of fruit- provided low salt whole meal bread bead, low salt margarine, and fruits	Blood pressure and lipids	Sodium restriction reduced blood pressure and did not raise low-density lipoprotein cholesterol. A low-fat/high-fibre diet did not reduce blood pressure but lowered cholesterol levels	?	?	Subjects followed either a low-sodium, low-fat/high-fibre diet (less than 60 mmol sodium/day; 30% fat energy; P:S ratio = 1; 30–50 g fibre/day) or a low-sodium, normal-fat/normal-fibre diet (less than 60 mmol sodium/day; 40% fat energy; P:S ratio = 0.3; 15 g fibre/day) for 8 weeks. Half of each group received 100 mmol/day NaC
1992	Anderson JW, Garrity TF, Wood CL, Whitis SE, Smith BM, Oeltgen A6PR. Prospective, randomised, controlled comparison of the effects of low- fat and low-fat plus high-fiber diets on serum lipid concentrations. Am J Clin Nutr. 1992;56:887–894	340 caucasian individuals between 30 and 50 y of age, and between 80% and I 20% of ideal body weight by the Metropolitan tables	Public screening of individuals drawn from major employers, churches, and shopping centers in the Central Kentucky area occurred between March and October of 1987	• l0-wk, diet-education program EDUCATIONAL SEMINAR protocols for the two diet groups were also quite similar and one instructor conducted the seminars for both groups - Dietary goals would be approached through a process of gradual change in habitual diet practices o 1-h sessions in which a modest amount of new information was presented each week; participant discussion and questions were encouraged. o Participants in both groups ( 10/group) were strongly encouraged to attend meetings with a spouse or close friend. o Demonstrations and active problem solving were major vehicles for learning. o Emphasis was placed on practical rather than theoretical knowledge (recipe modification, shopping strategies, label reading, and healthful restaurant eating were topics of seminar sessions) o Dietary-fat reduction was stressed for both diet groups• Appropriate instructional materials: some prepared by project staff and some available from the AHA and the HCF Nutrition Research Foundation (8). After the seminar, each participant and partner met individually for 30 mm with a consulting dietitian assigned to them for the duration of the project. o They discussed individual dietary progress and problems of the past week, o They set achievable goals for the next week. • Participants were encouraged to call the dietitian during the week when problems and questions arose with regard to the diet regimen. • These dietitians also made home visits 4 times during the year at which further instruction and problem solving were accomplished, as well as data collection integral to the diet assessment. • Each dietitian was responsible for four participants, two each from the two diet groups. • When a participant missed a seminar session, the participant was asked to attend the same session when it was offered during the subsequent wave of the study or individually with the dietitian	Lipids	The high-fiber group experienced a greater average reduction (13%) in serum cholesterol than did the low-fat (9%) and usual-diet (7%) groups. After adjustment for relevant covariates, the reduction in the high-fiber group was significantly greater than that in the low-fat group (P = 0.0482)	Instructor + consulting dietician	Educational seminar	AHA diet: • 55% of energy from carbohydrate, 20% of energy from protein, 25% of energy from fat, and 200 mg dietary cholesterol/d • amount of dietary fiber recommended, namely = 15 g • Preplanned meal patterns tailored to meet each individual’s lifestyle, preferences, and habits - three servings each of fruits and vegetables;—four servings of bread or starch foods;—two low-fat dairy items;—198.45 g lean meat, poultry, or seafood;—no egg yolk;—and fat servings based on energy content HCF diet: • 55% of energy from carbohydrate, 20% of energy from protein, 25% of energy from fat, and 200 mg dietary cholesterol/d. • amount of dietary fiber recommended = 50 g at least one serving of beans and one serving of cereal chosen from the HCF exchange groups • The use of soluble-fiber rich cereals such as oat bran was encouraged
1994	Campbell, M.K., DeVellis, B.M., Strecher, V.J., Ammerman, A.S., DeVellis, R.F., Sandler, R.S. Improving dietary behavior (the effectiveness of tailored messages in primary care settings). Am J Public Health. 1994;84:783–787	558 adult patients (ages 18 and above) recruited from four family practices in central North Carolina between September and November 1991	primary care, Two practices served a primarily urban population and two were primarily rural	The tailored intervention consisted of a one-time, mailed nutrition information packet tailored to the participant's stage of change, dietary intake, and psychosocial information. All tailored group members were mailed a packet containing a nutrition profile summarizing their current diet and level of interest in changing behavior, a tailored page regarding dietary fat intake, and a tailored page regarding fruit and vegetable intake. Each message acknowledged the participant's stage and addressed his or her beliefs about both susceptibility to diet-related diseases and perceived benefits of and motives for changing diet. Individualized diet feedback was then provided regarding baseline fat and fruit/vegetable intake. Contemplators received information designed to decrease barriers to change and to increase self-efficacy. Depending on stage of change, self-efficacy, and history of past relapse, participants also received tailored recipes and specific diet tips designed to promote skills and to provide cues to action. Those individuals who were already trying to change received tailored recipes and messages aimed at preventing relapse	Change of dietary habits	Tailored nutrition messages are effective in promoting dietary fat reduction for disease prevention		tailored mailed nutrition information	1990 Dietary Guidelines for Americans
1996	Siggaard R, Raben A, Astrup A. Weight loss during 12 weeks ad libitum carbohydrate-rich diet in overweight and normal weight subjects at a Danish working site. Obes Res 1996; 4:347 ± 356	86 normal-weight and overweight employees	Danish worksite	Before Intervention: 4-day dietary record based upon weighing. Weighed and instructed weekly in how to achieve and maintain an ad libitum carbohydrate- rich, low-fat diet. The instructions were a combination of lectures and written material. The material contained information on different topics related to nutrition, e.g. physiological mechanisms involved in appetite regulation and macronutrient balance, the macronutrient content of different food, the importance of carbohydrate-rich, low-fat snacks and how to read food labels. The dietary guideline contained carbohydrate-rich and low-fat recipes (8 recipes for breakfast, 12 for lunch, 6 for snacks and 55 for dinner). The subjects were encouraged to choose some of the recipes for their meals and were told that they could eat as much as they wished until satisfaction	Weight loss	a significant loss of body weight (4.2 ± 0.4 kg) and fat mass (4.4 ± 0.6 kg) was observed in I (p < 0.05 vs. C). The weight loss in I was not regained at 24 and 52 weeks' follow-up (82% of I participating) compared to baseline	?	Lectures + written material	The emphasis was placed on the consumption of natural high-carbohydrate foods such as whole grains, fruits, vegetables and low-fat dairy products, but there were no limitations as to the source of carbohydrate Patients were given a dietary guideline developed to increase the daily carbohydrate intake and decrease the daily fat intake. The guideline guaranteed a minimum intake of 7.0 to 8.0 MJ/d with carbohydrate amounting to 60 to 65 E%, protein to 15 to 20 E%, fat to 20 to 25 E% and dietary fiber to 4.9 to 6.2 glMJ
1997	Beresford, S.A., Curry, S.J., Kristal, A.R., Lazovich, D., Feng, Z., Wagner, E.H. A dietary intervention in primary care practice (the Eating Patterns Study). Am J Public Health. 1997;87:610–616	2111 patients of six primary care clinics		The intervention consisted of two components: a self-help booklet and physician endorsement. We developed the booklet, Help Yourself: a guide to healthful eating, on the basis of behavior change principes derived from the social learning theory, and the dietary recommendations of the National Research Council. We presented motivations for dietary change such as improving health, following the changing social norm to eat lower fat, higher fiber foods, and doing something positive for oneself. Current dietary behavior was assessed trough the use of a brief self test at the beginning of the book. We presented specific behaviors skills in an easy to follow format, beginning by identifying current behaviors and suggesting sequential changes in small, simple steps. No external goals were included: rather, individuals were encouraged to set their own goals	Change of dietary habits	Intervention and control groups both reported a decrease in fat intake and an increase in fiber intake. The differential change and 95% confidence interval (CI) for the percentage of energy obtained from fat was -1.2 (CI = -0.71, -1.7) (P = .0015), for grams fiber/1000 kcal 0.32 (CI = -0.066, 0.71) (P = .086), for fat score -0.044 (CI = -0.016, -0.072) (P = .010), and for fiber score 0.036 (CI = 0.011, 0.061) (P = .014)	book + physician	information	healthy eating
1997	Glasgow RE, La Chance PA, Toobert DJ, Brown J, Hampson SE, Riddle MC. Long-term effects and costs of brief behavioural dietary intervention for patients with diabetes delivered from the medical office. Patient Educ Couns 1997; 32: 175–84	206 Diabetic patients > 40 yo, in primary care		Before: 4-day food record form and questionnaire. A 5–10 min touchscreen dietary barriers assessment (Barriers to dietary self-care are an expanded list of 30 questions adapted from the Barriers to SelfCare instrument that immediately generated two printed feedback forms:—for the patient—for the physician with information on four issues: 1. question the patient would most like to discuss at that visit (reported verbatim from patient statements) 2. the patient's dietary intake 3. summary of the key barriers 4. a visual analog scale indicating the patient's standing on: readiness to engage in dietary change, seriousness of diabetes, importance of diabetes, desire for participation in decisions. Then: Brief (20-min) patient centered goals, setting and problem solving, and to receive dietary self-help materials coordinated with the computer feedback, to lower fat intake. Patient with higher self-efficacy estimated level: home video adressing strategies for the most frequent type of barriers. Patient with lower self-efficacy estimated level: returned for 30 min individuaized video via a touch screen. Follow-up by phone at weeks 1, 3 and at 3 and 6 months: review, adjust strategies, discuss difficulties, give more material. Book "The Human Side of Diabetes" was given at 9 months	Change of dietary habits + Lipids	Brief Intervention produced significantly greater improvement than Usual Care on multiple measures of change in dietary behaviour (e.g., covariate adjusted difference of 2.2% of calories from fat; p = 0.023) and on serum cholesterol levels (covariate adjusted difference of 15 mg/dl; p = 0.002) at 12-month follow-up. There were also significant differences favouring intervention on patient satisfaction (p < 0.02) but not on HbA1c levels. The costs of intervention ($137 per patient) were modest relative to many commonly used practices	physician + follow-up by phone	4-day food record and dietary validated questionnaire = book at 9 month	the Human side of diabetes
1997	Keyserling, T.C., Ammerman, A.S., Davis, C.E., Mok, M.C., Garrett, J., Simpson, R.J. A randomized controlled trial of a physician-directed treatment program for low-income patients with high blood cholesterol (the Southeast Cholesterol Project). Arch Fam Med. 1997;6:135–145	372 low-income and minority patients with high cholesterol	Twenty-one community and rural health centers in North Carolina and Virginia	3 major components paralleling the NCEP recommendations: (1) a clinician-directed dietary component using the Food for Heart Program (FFHP); (2) referral to a local dietitian if LDL-C remained elevated at 4-month follow-up (3) a prompt for the clinician to consider drug treatment based on the LDL-C at 7-month follow-up In addition, a quarterly reinforcement mailing with recipes and health tips was sent to all intervention patients after they returned for their 7-month blood test Food for Heart Program which consists of the following components: (1) The dietary risk assessment (DRA), a validated food-frequency instrument that identifies major sources of saturated fat and cholesterol in the diet (2) A color- and number-coded educational strategy that guides clinician counseling without requiring extensive knowledge of behavior-change theory or food composition (3) Easy-to-read, illustrated patient education materials that are culturally specific to the population served, promote interaction between patient and clinician, divide recommendations into small achievable steps, and offer practical assistance for dietary change (4) record goals and monitor patient progress Dietitian Referral If the LDL-C remained elevated at 4 months, participants were referred to a dietitian or health educator for a maximum of 3 counseling sessions, each lasting 30 min. They were trained to use the FFHP educational materials in greater depth and to supplement this program with other materials as appropriate. At the end of 3 counseling sessions, the dietitians or health educators completed a summary checklist that was mailed to the clinic and filed in the patient's FFHP folder. This checklist served as feedback to the clinician and as a guide for long-term monitoring and reinforcement at subsequent clinic visits Prompt for Use of Lipid-Lowering Medication The prompt consisted of a letter for the clinician and a drug treatment folder for the patient's chart. The folder included a quick overview of NCEP guidelines for initiating drug therapy, including medications of choice and their cost, detailed information on each class of lipid-lowering agents and a flow diagram illustrating how to use the agents, and simply written, single-page handouts for the patient describing the importance of each medication, getting started, increasing the dosage, coping with potential adverse effects, and other information designed to help maximize adherence to the medical regimen TRAINING: A nutritionist on the study staff trained intervention clinicians to use the FFHP during a 90-min tutorial that included a brief review of essential elements of a lipid lowering diet, dietary behavior-change strategies, use of the FFHP materials, and practice using the materials	Lipids	Total cholesterol and LDL-C decreased more in the intervention group than in the control group. Overall, the difference in lipid reduction between groups was modest and of borderline statistical significance; among participants who did not take lipid-lowering medication during follow-up, the difference in lipid reduction between groups was larger	dietician	educational program lead by dietician	NCEP recommendations and FFHP educational material
1997	Roderick, P., Ruddock, V., Hunt, P., Miller, G. A randomized trial to evaluate the effectiveness of dietary advice by practice nurses in lowering diet-related coronary heart disease risk. Br J Gen Pract. 1997;47:7–12	956 participants aged 35–59 years, recruited opportunistically by their GPs	primary care	**Trained NURSES** nurses were trained by a state registered dietician over a one-and-a-half-day period in diet and its relationship with coronary heart disease, how to use the nutritional tools (including interpretation of the food frequency questionnaire to assess dietary habits), and how to negotiate dietary changes with each patient **Intervetion** All: standard health education from the leaflets Guide to Healthy Eating, Giving up smoking, Look After Your Heart, Heart Disease, and Exercise, Why Bother? Intervention: o Negociating changes, food substitution (objectives: 5 changes), review of quantity/freqency food intake o Specially designed dietary sheets: all foods were classified as ‘to eat plentifully’, ‘in moderation’ or ‘on special occasions only’ o Special leaflets o Patients who were overweight (BMI over 25 kg/m2) were given special advice, including a self-monitoring chart and a choice of a calorie-restricted diet o Diet: reduction in total and saturated fat intake and an increased consumption of complex carbohydrates and fruit and vegetables o Changes recorded by nurse **Follow UP** 4–6 weeks, at which time progress with dietary change was assessed, weight remeasured and further changes agreed if appropriate High CVD risk or High CHolesterol– > furthre follow up at 3 and 6 months	Lipids + Weight loss	Compliance with annual follow up was 80%. Compared with 'usual care' practices, there was a mean 0.20 mmol/l lower serum cholesterol (95% CI -0.38 to -0.03 at 1 year) in 'dietary advice' practices. There was a small fall in weight of 0.56 kg (95% CI -1.04 to -0.07) and reductions in total and saturated fat. Factor VII coagulant activity fell by a mean of 6.7% of the standard (95% CI -15.4 to + 2.0)	nurses trained by dietician	negociating changes	for overweighted patients a choice of a calorie-restricted diet o Diet: reduction in total and saturated fat intake and an increased consumption of complex carbohydrates and fruit and vegetables
1999	Fleming MF, Manwell LB, Barry KL, Adams W, Stauffacher EA. Brief physician advice for alcohol problems in older adults: a randomized community-based trial. J Fam Pract 1999; 48: 378–84	105 men and 53 women. alcohol drinkers > 14 alcoholic drinks/wk (11 drinks/wk for women)	primary care, Twenty-four community-based primary care practices in Wisconsin	Intervention group patients received two 10- to 15-min physician-delivered counseling sessions that included advice, education, and contracting using a scripted workbook. Patients randomized to the intervention group were given the same booklet than the control group (general health advice) and were scheduled to see their personal physicians. The brief intervention protocol used by the participating physician included a workbook containing feedback on the patient's health behaviors, a review of problem-drinking prevalence, reasons for drinking, adverse effects of alcohol, drinking cues, a drinking agreement in the form of a prescription, and drinking diary cards. Two 10- to 15-min visits with the physician were scheduled 1 month apart (a brief intervention and a reinforcement session). Each patient received a follow-up phone call from the clinic nurse 2 weeks after each visit. Patient follow-up procedures included telephone interviews at 3, 6, and 12 months. Family members were contacted at 12 months to corroborate the patients' self-reports	alcohol consumption	At 12 months, There was a 34% reduction in 7-day alcohol use, 74% reduction in mean number of binge-drinking episodes, and 62% reduction in the percentage of older adults drinking more than 21 drinks per week in the intervention group compared with the control group	Trained physicians	Brief advice. Intervention group patients received two 10- to 15-min physician-delivered counseling sessions that included advice, education, and contracting using a scripted workbook	no
1999	Lutz, S.F., Ammerman, A.S., Atwood, J.R., Campbell, M.K., DeVellis, R.F., Rosamond, W.D. Innovative newsletter interventions improve fruit and vegetable consumption in healthy adults. J Am Diet Assoc. 1999;99:705–709	710 health maintenance organization clients	Healthy adults population	3 intervention groups receiving: 2) non tailored newsletters, 3) computer-tailored newsletters: Participants' responses to the baseline survey were used to create messages for the tailored newsletters. Fruit or vegetable information used to tailor each newsletter included intake, eating behaviors (eg, frequency that fruits and vegetables are eaten as a snack), nutrition-related activities (eg, cooking and shopping), and psychosocial factors (eg, perceived barriers) 4) tailored newsletters with tailored goal-setting information Intervention groups received 1 newsletter each month for 4 months The baseline survey were used to tailor the newsletters Tailored goal-setting messages: subjects were given the specific difficult goal of "increasing fruit and vegetable intake to 5 or more servings each day." VS a vague, nonquantitative goal of "eating more fruits and vegetables." + 3 tailored subgoals to help them achieve the overall goal of "5 a day." These subgoals were selected using participants' responses to the eating behavior questions from the baseline survey	Change of dietary habits	food frequency questionnaire		Theoretical constructs: self-efficacy from the Social Cognitive Theory, stage of readiness to change from the Transtheoretical Model of Change, and perceived barriers and benefits from the Health Belief Model + Goal-setting theory guided development of the tailored newsletters with a goal-setting component	All newsletters contained strategies for improving fruit and vegetable consumption. Tailored newsletters used computer algorithms to match a person's baseline survey information with the most relevant newsletter messages for promoting dietary change
1999	Ockene IS, Hebert JR, Ockene JK, et al. Effect of physician-delivered nutrition counseling training and an office-support program on saturated fat intake, weight, and serum lipid measurements in a hyperlipidemic population: Worcester Area Trial for Counseling in Hyperlipidemia (WATCH). Arch Intern Med. 1999;159:725–31	Eleven hundred sixty-two patients: 20–65 y/o, no prior lipid-lowering drug treatment, no dietitian referral within 1 yea	Primary care. Forty-five primary care internists at the Fallon Community Health Plan	Interventions: - 8 to 10 min interview with PCP - WATCH intervention: o one-on-one counseling and attend group sessions o 1) increase patients' awareness of the risk factors associated with coronary heart disease; o 2) provide patients with nutrition knowledge to promote the lowering of blood cholesterol levels; o 3) increase patients' confidence in their ability to make dietary changes; o 4) enhance patient's skills needed for long-term changes in eating patterns Training: Primary care physicians o 2 sessions: § 2,5 h small group sessions (3–10 physicians): didactic instruction, videotape observation, and role-playing § 30’ individualized tutorial: role-playing the counseling-intervention approach with a patient simulator • 1) advise nutrition change and use personalized information to reinforce the need for such change; • (2) assess experience with dietary change to determine the patient's resources for change (strengths) and factors that inhibit it (barriers); • (3) review current diet using the Dietary Risk Assessment • (4) prioritize areas of high-fat intake; • (5) develop a plan for change; and • (6) arrange for follow-up - Office-support program: no training, series of handouts to be given to the patient, including the Dietary Risk Assessment, goal sheets containing dietary change recommendations, a brief cooking and recipe guide, tips for eating out, motivational material, and suggestions for further reading	Change of dietary habits	Effective for the third group (+ office support). Compared with group 1, patients in group 3 had average reductions of 1.1 percentage points in percent of energy from saturated fat (a 10.3% decrease) (P = .01); a reduction in weight of 2.3 kg (P < .001); and a decrease of 0.10 mmol/L (3.8 mg/dL) in low-density lipoprotein cholesterol level (P = .10). Average time for the initial counseling intervention in group 3 was 8.2 min, 5.5 min more than in the control group	primary care physicians	WATCH intervention	changes adapted to the Diaetary risk assessment
2000	Kristal, A.R., Curry, S.J., Shattuck, A.L., Feng, Z., Li, S. A randomized trial of a tailored, self-help dietary intervention (the Puget Sound Eating Patterns study). Prev Med. 2000;31:380–389	Adults over 18 who were enrolled in the Group Health Cooperative of Puget Sound	home-based and health care facility-based	Participants received a package of self-help materials, dietary analysis with behavioral feedback and a semi-monthly newsletter. The self-help packet included an introductory letter with computer generated messages, a “Help Yourself” manual with inform ation, suggestions, skills, stickers and handwritten notes, individualized dietary change materials such as tip sheets, refrigerator magnet, recipe cards, shopping lists and self evaluations, and computer generated behavioral feedback. Participants received a motivational phone call. Providers received training for incorporating the self-help intervention into the medical visit. Just prior to the appointment, a self-help booklet and a script to introduce it were placedin the patient's medical chart. Telephone based surveys measured change in fat intake, food frequency, stages of change, and dietary recalls. Patients participated in a cholesterol screening Budget: $57 per patient Intervention: Self-help kit, newsletters, letters, manuals, tip sheets, magnets, recipes, shopping lists, self-evaluations, telephone, motivational call script Evaluation: Surveys, cholesterol screening device, telephone	Change of dietary habits	The intervention effect ± SE for fat, based on a diet habits questionnaire, was -0.10 ± 0.02 (P < 0.001), corresponding to a reduction of approximately 0.8 percentage points of percentage energy from fat. For fruits and vegetables, the intervention effect was 0.47 ± 0.10 servings/day (P < 0.001)	?	computer, telephone and self-help intervention	?
2000	Sasaki S, Ishikawa T, Yanagibori R, Amano K. Change and 1-year maintenance of nutrient and food group intakes at a 12-week worksite dietary intervention trial for men at high risk of coronary heart disease. J Nutr Sci Vitaminol 2000;46(1):15–22	320 workers of Toho-Gas Compagny, Nogoya, Japan, with nonfasting serum cholesterol, nonfasting blood glucose, and/or BMI > 25 at the annual health checkup	annual health checkup	Using the pre-intervention dietary assessment: an individual results sheet used in the education consisted of 7 pages with a summary of the subject's dietary habits and individualized advice for dietary modification Approximately 15 min of individual counseling was performed by trained nurses under the supervision of a registered dietitian During the subsequent 12wk, a newsletter was sent to the subjects every week. The newsletter consisted of an easy-to-understanding story on "how to normalize your serum cholesterol level, body weight, or blood glucose by modifying your diet," or related topics for cardiovascular prevention. The education method used in the study is described in detail more elsewhere	Change of dietary habits	The Keys score, and the changes in intake of saturated fatty acids (SFA), monounsaturated fatty acid, total fat, and cholesterol (the decrease), as well as dietary fiber, potassium, calcium, and iron (the increase) were significantly different between the intervention (n = 63) and control (n = 123) groups (p < 0.05). The changes were almost maintained with little recidivism at the 1 y follow-up point in the intervention group (i.e., for the decrease in SFA and Keys score, p < 0.001)	trained nurses supervised by dietician	counselling +	?
2000	Siero, F.W., Broer, J., Bemelmans, W.J., Meyboom-de Jong, B.M. Impact of group nutrition education and surplus value of Prochaska-based stage-matched information on health-related cognitions and on Mediterranean nutrition behavior. Health Educ Res. 2000;15:635–647	252 persons with three risk factors for development of cardiovascular disease: serum cholesterol between 6 and 8 mmol/l was needed as well as two or more of the following CVD risk factors: high blood pressure, a BMI above 27 kg/m2, smoking, diagnosis of CVD or a first-degree relative with a history of CVD before the age of 60 years	inhabitants older than 30 years old of age of two counties within a region with a high CVD mortality received a written invitation to participate in a screening program for blood pressure. The study region is a socio-economically deprived area in the Netherlands	The first intervention consisted of three meetings in which the positive health effects of a Mediterranean diet were discussed in group sessions. In the additional intervention stage-matched information based on the Transtheoretical Model of behavior change was given. Both intervention groups were compared with a control group, which received only a printed leaflet with the Dutch nutritional guidelines	Change of dietary habits	improvement of diet	?	behavioral change, group sessions	Mediterranean diet
2000	Wardle J, Rogers P, Judd P, Taylor MA, Rapoport L, Green M, et al. Randomized trial of the effects of cholesterol-lowering dietary treatment on psychological function. American Journal of Medicine 2000;108(7):547–53	176 adults with mildly or moderately raised serum cholesterol levels	hospital dietetic clinics, hospital physicians, and general practitioners in London and Southeast England	Low fat: shift away from foods containing saturated fats, with a target to reduce energy from fats to < 20%, largely polyunsaturates Med diet: increase in fruit and vegetables, an increase in oily fish, and a reduction in fat to 30% of energy, with substitution of predominantly monounsaturated fats for saturated fats 12-week period using a combination of individual and group sessions with a dietitian and a psychologist Content: education about the recommended dietary changes and a cognitive-behavioral intervention that was concerned with implementing changes in eating behavior – > individualized advice on implementing the dietary changes based on their lifestyle and food preferences, – > and group support in maintaining changes They were also given free-spreading fats and oils that were high in polyunsaturated fat (low-fat diet) or monounsaturated fat (Mediterranean diet) to encourage compliance	Lipids	Two dietary interventions that successfully lowered serum cholesterol levels had no adverse effect on mood	dietician + psycholoist	education and cognitive behavioral intervention groups	Low fat diet and MED diet
2001	Delichatsios, H.K., Friedman, R.H., Glanz, K. et al., Randomized trial of a “talking computer” to improve adults’ eating habits. Am J Health Promot. 2001;15:215–224	298 sedentary participants with suboptimal diet quality. To be eligible for participation in the study, individuals needed to be sedentary and have a "suboptimal" diet, Individuals were excluded if they reported engaging in regular moderate or vigorous physical activity or regular vigorous physical activity	primary care	Interactive computer-based system called Telephone-Linked Communications (TLC):an at-home monitor, educator, and counselor for individuals interested in changing health-related behaviors The patients’ physicians received reports so they also could be aware of their patients’ progress Using computer-mediated digitized human speech over the telephone, TLC asks questions to monitor the patient’s behaviors and health conditions and provides education and behavioral reinforcement for targeted health-related behaviors such as medication-taking, adherence to diet, regular physical activity, and cigarette smoking cessation The TLC-Eat use social cognitive theory as the guide to behavior change and a multidimensional approach to monitor and intervene upon dietary behaviors, including attention to (1) food consumption, (2) food knowledge, (3) food purchasing, (4) preparation and cooking, (5) food serving and garnishing, and (6) restaurant food selection The TLC-Eat intervention focuses on intrapersonal factors by linking the behavior of eating to personally valued outcomes, by enhancing the person’s sense of successfully modifying behavior, and by providing both positive and corrective reinforcement for continual and incremental change in behavior and prevention of relapse For example, TLC-Eat asks a series of food consumption questions to assess recent intake in a food category Next, TLC-Eat issues a friendly challenge, "How about between now and your next call that you try to eat 3 fruits a day? Are you up for the challenge?" The user can press 1, signaling "yes," or 2 for "no." If yes, TLC-Eat replies, "Okay, next time we talk, we’ll see how you are doing." If the user responds "no," TLC-Eat will negotiate the user’s goal for fruit consumption, following the precepts of shared decision-making We instructed participants to call the system once a week for 6 months (26 calls). If they were late in calling by 2 weeks, they received reminder calls from the TLC system	Change of dietary habits	Daily intake of fruits, vegetables, red and processed meats, whole fat dairy foods, and whole grain foods estimated from a food frequency questionnaire	Interactive computer-based system called Telephone-Linked Communications (TLC):an at-home monitor, educator, and counselor for individuals interested in changing health-related behaviors	Interactive computer-based system called Telephone-Linked Communications (TLC):an at-home monitor, educator, and counselor for individuals interested in changing health-related behaviors	TLC-Eat focuses on fruits, vegetables, red and processed meats, whole fat dairy foods, and whole grain foods
2001	Delichatsios, H.K., Hunt, M.K., Lobb, R., Emmons, K., Gillman, M.W. EatSmart (efficacy of a multifaceted preventive nutrition intervention in clinical practice). Prev Med. 2001;33:91–98	504 participants from primary care practices	primary care, at home	Diet education, promotion by motivational consultations and communication tools,Combined electronic and phone intervention, primary care giver reinforcement, mails, diet, behavioral strategies (MI) three main components to the intervention: • Personalized letter for each of the target food groups at baseline based on subjects’ consumption as reported on the initial assessment; • In the letter we suggested what action the participants should take (increase, decrease, or continue the same) for the food groups according to the dietary standards for the study: ~ 5 daily servings of fruits and vegetables; ~ 2 daily servings of low-fat dairy products; ~ 2 weekly servings of whole-fat dairy products; ~ 3 weekly servings of red and processed meats) • stage-matched educational booklets by mail: information on food selection, preparation, and behavior change • (2) GP endorsement of the recommendations, • (3) two motivational counseling sessions by telephone • A consultation with the study nutritionist was available• • We asked providers to focus their endorsement on one food group based on patients’ preferences and need for improvement o At the minimum, we asked that they stress that diet is an integral part of a healthful lifestyle o These recommendations were summarized on a laminated card for easy access during the patient encounter • We had several safeguards in place to improve the chance that the endorsement would occur • trained telephone counselors conducted 2 motivational counseling sessions over the telephone 2 weeks and 2 months after the provider visit o modeled on the technique of motivational interviewing, a directive, client-centered counseling style that enhances motivation for change by helping the client clarify and resolve ambivalence about behavior change o telephone counselors encouraged participants to set dietary goals and assisted participants with identifying strategies to achieve these goals o The telephone counselors incorporated the participants’ stage of change in the messages of the motivational counseling sessions, o The telephone counselors used the ADA Food and Nutrition Guide as a reference during the sessions and mailed out information and additional study nutrition booklets as participants requested them o If nutrition questions arose during the motivational counseling sessions that the telephone counselors felt needed further expertise, they referred these subjects to the study nutritionist Training of care givers: • A study nutritionist • PCPs were either primary care physicians or nurse practitioners . 1-h training session for PCPs at each intervention site during a regularly scheduled meeting . We provided a videotape for the PCPs who did not attend the training • Trained telephone counselors conducted two motivational counseling sessions o The three telephone counselors were Master’s level public health students and they attended two 4-h training sessions on motivational counseling techniques and a 2-h training on foods and nutrients related to the primary outcomes o Weekly meetings of the health educator team during the intervention period allowed for ongoing supervision and evaluation of the motivational counseling sessions	Change of dietary habits	We measured change in intake of foods using results from the baseline and follow-up food frequency questionnaires	diet education, promotion by motivational consultations and communication tools,Combined electronic and phone intervention, primary care giver reinforcement, mails, diet, behavioral strategies (MI)	behavioral strategies (MI)	ADA Food and Nutrition Guide • In the letter we suggested what action the participants should take (increase, decrease, or continue the same) for the food groups according to the dietary standards for the study: ~ 5 daily servings of fruits and vegetables; ~ 2 daily servings of low-fat dairy products; ~ 2 weekly servings of whole-fat dairy ~ 3 weekly servings of red and processed meats) products;
2003	Appel LJ, Champagne CM, Harsha DW, et al. Effects of comprehensive lifestyle modification on blood pressure control: Main results of the PREMIER clinical trial. JAMA. 2003;289(16):2083–2093	810 adults with optimal BP, stage 1 hypertension (120–159 mm Hg systolic and 80–95 mm Hg diastolic), and not taking antihypertensive medications	Ok	An interventionist (dietitian) discussed nonpharmacological factors that affect BP and provided printed educational materials. A single 30-min individual session. Counseling on behavior change was not provided. No further contact with the interventionist occurred until after completion of the data collection visits at 6 months DASH intervention: increased consumption of fruits and vegetables (9–12 servings/d) and low-fat dairy products (2–3 servings/d), and reduced intake of saturated fat (≤ 7% of energy) and total fat (≤ 25% of energy) During the initial 6 months, there were 18 face-to-face intervention contacts (14 group meetings and 4 individual counseling sessions). Participants kept food diaries, recorded physical activity, and monitored calorie and sodium intake. Monitored intake of fruits, vegetables, and dairy products and monitored their intake of fat	blood pressure	Blood pressure measurement and hypertension status at 6 months	Dietician	there were 18 face-to-face intervention contacts (14 group meetings and 4 individual counseling sessions)	DASH diet and LNAHK diet
2003	Foster GD, Wyatt HR, Hill JO et al. A randomized trial of a low-carbohydrate diet for obesity. N Engl J Med 2003; 348: 2082–2090	63 subjects		Atkinslow carbohydrate diet VS low calories conventional diet, Low carbohydrate diet Material: A copy of Dr. Atkins' New Diet Revolution was provided Conventional diet: • A copy of The LEARN Program for Weight Management which provides 16 lessons covering various aspects of weight control. The nutritional information in the manual was consistent with the dietary recommendations provided by the study dietitian and with the Department of Agriculture Food Guide Pyramid • Subjects were instructed to read the manual and follow the program as described Led by Dietitian	Weight loss	Subjects on the low-carbohydrate diet had lost more weight than subjects on the conventional diet at 3 months (mean [± SD], -6.8 ± 5.0 vs. -2.7 ± 3.7 percent of body weight; P = 0.001) and 6 months (-7.0 ± 6.5 vs. -3.2 ± 5.6 percent of body weight, P = 0.02), but the difference at 12 months was not significant (-4.4 ± 6.7 vs. -2.5 ± 6.3 percent of body weight, P = 0.26)	Dietician	First meeting with dietician. Atkins diet: Each subject was given a copy of Dr. Atkins' New Diet Revolution, 10 which details the Atkins diet program. Subjects were instructed to read the book and follow the diet as described. Conventional diet: Subjects were given a copy of The LEARN Program for Weight Management, 17 which provides 16 lessons covering various aspects of weight control	Low carbohydrate diet • low-carbohydrate, high-protein, high-fat diet met • individual consultation with a registered dietitian before beginning the program • The Atkins diet is organized into four phases: induction, ongoing weight loss, premaintenance, and maintenance • limiting carbohydrate intake without restricting consumption of fat and protein • For the first two weeks, carbohydrate intake is limited to 20 g per day and is then gradually increased until a stable and desired weight is achieved. Conventional diet: • conventional diet: a high-carbohydrate, low-fat, low-calorie diet o Calories reduction: 1200 to 1500 kcal per day for women and 1500 to 1800 kcal per day for men, o with approximately 60 percent of calories from carbohydrate, 25 percent from fat, and 15 percent from protein) o calorie counting • individual consultation with a registered dietitian before beginning
2004	Carpenter RA,.Finley C. Pilot test of a behavioral skill building intervention to improve overall diet quality. Journal of Nutrition Education & Behavior 2004;36:20–4	98 generally healthy participants; men (n = 35) and women (n = 63); mean age = 49.6 years (range = 29 to 71 years)	primary care	Curriculum strategy: cognitive strategies: increasing knowledge, warning of risks, caring about consequences to others, comprehending benefits, increasing healthful opportunities: behavorial alternatives: enlisting social support, substituting alternatives, rewarding yourself, commiting yourself, reminding yourself All 3 groups received a copy of The American Dietetic Association’s Complete Food & Nutrition Guide and instructions to contact study staff by telephone or electronic mail if they had any questions Meeting met in small groups (n = 13 to 15 people) with 2 staff co-facilitators weekly for the first 16 weeks and biweekly for the last 8 weeks, for a total of 20 sessions The 75-min sessions included a brief round robin check-in, presentation of the session topic and review of session materials, interactive learning strategies to personalize the topic to participants’ respective lives, review of the home assignment, and preview of the next session. Participants were encouraged to turn in weekly food logs for feedback	Change of dietary habits	improvement of the Modified Healthy Eating Index	?	cognitive strategies in group sessions Transtheoretical Model12 and Social Cognitive Theory.13 Table 1 shows how we incorporated strategies from the Transtheoretical Model into our curriculum.The constructs of self-efficacy, self-regulation, and expectancies from the Social Cognitive Theory were built into the curriculum by promoting small changes, frequent weighing of benefits and barriers of changing dietary habits, setting short-term goals and rewards, developing relapse prevention strategies, and identifying ways to making healthful eating fun	American Dietetic Association’s Complete Food & Nutrition Guide
2004	Stern L, Iqbal N, Seshadri Pet al. The effects of low-carbohydrate versus conventional weight loss diets in severely obese adults: one-year follow-up of a randomized trial. Ann Intern Med 2004; 140: 778–785	132 participants, 18 years of age and older with a body mass index (BMI) of 35 kg/m2 or greater	outpatient practices of the Philadelphia Veterans Affairs Medical Center. 132 obese adults with a body mass index of 35 kg/m2 or greater; 83% had diabetes or the metabolic syndrome	Low carbohydrate vs Low calorie low fat diet: - two-hour group-teaching sessions each week for four weeks, • Material: o Subjects received a diet-overview handout, o instructional nutrition labels, o sample menus and recipes, and o a book on counting calories and carbohydrates Follow up: followed by monthly one-hour sessions for five additional months	Weight loss + Lipids + glycemic control	By 1 year, mean (± SD) weight change for persons on the low-carbohydrate diet was -5.1 ± 8.7 kg compared with -3.1 ± 8.4 kg for persons on the conventional diet. Differences between groups were not significant (-1.9 kg [95% CI, -4.9 to 1.0 kg]; P = 0.20). For persons on the low-carbohydrate diet, triglyceride levels decreased more (P = 0.044) and high-density lipoprotein cholesterol levels decreased less (P = 0.025). As seen in the small group of persons with diabetes (n = 54) and after adjustment for covariates, hemoglobin A1c levels improved more for persons on the low-carbohydrate diet. Droup out 34%	experts in nutritional counseling	diet-overview handout, 2 h group sessions weekly for 4 week, one hour monthly session for 5 months	Participants on the low-carbohydrate diet were instructed only to reduce carbohydrate intake to less than 30 g per day. Participants on the conventional diet were instructed to reduce caloric intake by 500 cal per day, with less than 30% of calories derived from fat, in accordance with the National Heart, Lung, and Blood Institute guidelines
2005	Azadbakht L, Mirmiran P, Esmaillzadeh A, Azizi T, Azizi F. Beneficial effects of a Dietary Approaches to Stop Hypertension eating plan on features of the metabolic syndrome. Diabetes Care. 2005;28(12): 2823–2831	116 patients with metabolic syndrome		DASH 500 kcal less than the participants caloric needs The patients had been visited monthly; each session for a patient was 45–60 min. Individual and group sessions They were in touch with the nutritionist by phone every day Behavioral and psychological counseling was offered The nutritionist explained the benefits of each diet for patients and told them if they continued these diets, related metabolic abnormalities might be controlled group discussions were conducted monthly First determined the caloric needs for each person – > The diets were individually prescribed using a calorie count system, and an exchange list was given to each patient for exchanging food items and counting the calories Self monitoring: Subjects were asked to write food diaries– > used for feedback at monthly meeting	Lipids + Blood pressure	Relative to the control diet, the DASH diet resulted in higher HDL cholesterol (7 and 10 mg/dl), lower triglycerides (-18 and -14 mg/dl), systolic blood pressure (SBP) (-12 and -11 mmHg), diastolic blood pressure (-6 and -7 mmHg), weight (-16 and -14 kg), fasting blood glucose (FBG) (-15 and -8 mg/dl), and weight (-16 and -15 kg), among men and women, respectively (all P < 0.001). The net reduction in triglycerides (-17 and -18 mg/dl), SBP (-11 and -11 mmHg), diastolic blood pressure (-5 and -6 mmHg), and FBG (-4 and -6 mg/dl), weight (-16 and -15 kg), and increase in HDL (5 and 10 mg/dl) among men and women, respectively, was higher in the DASH group (all P < 0.05). The weight-reducing diet resulted in significant change in triglycerides (-13 and -10 mg/dl), SBP (-6 and -6 mmHg), and weight (-13 and -12 kg) among men and women, respectively (all P < 0.05)	Nutritionnist	Individual and group sessions. Behavioral and psychological counseling was offered	DASH 500 kcal less than the participants caloric needs, which was increased in fruit, vegetables, and low-fat dairy products and lower in saturated fat, total fat, and cholesterol, containing more whole grains and fewer refined grains, sweets, and red meat. The calcium, potassium, and magnesium of the DASH diet were higher. The DASH diet contained 2,400 mg Na per day. Lower consumption of meat and higher consumption of low-fat dairies, vegetable, fruit, whole-grain cereals, and legumes distinguish between the DASH trial and the weight-reduction diet
2005	Cook NR, Kumanyika SK, Cutler JA, Whelton PK. Dose–response of sodium excretion and blood pressure change among overweight, nonhypertensive adults in a 3-year dietary intervention study. J Hum Hypertens 2005;19:47–54	1154 healthy men and women ages 30–54 years, ages 30–54 years, body weight 110–165% of sex-specific standard weight	community, at nine clinical centres located in diverse regions of the United States, with a coordinating centre located in Boston	Meeting types: an initial in person individual meeting, 10 weekly group meetings, four monthly group meetings and additional less frequent group sessions for the remainder of follow-up. Individual telephone and face-to-face contacts were offered as needed to facilitate maximum adherence Intervention: Sodium reduction: first on morning meals where food choices are relatively limited, and then progressing through the day to noontime meals, evening meals, and snacks Finally, the options for eating out were considered, planning, reading labels, shopping, and modifying recipes - sodium knowledge: to identify sources of sodium in their food - building behavioral skills to control sodium intake in a variety of situations - encouragement to explore a variety of foods and flavors - Sampling of products and recipes exposed participants to the range of interesting seasonings that are possible with low-sodium foods and made them aware of the many available options - emphasis on enjoyment of food - articles describing sodium as an effective intervention for reducing blood pressure of hypertensives were discussed during sessions, to emphasize the potential benefits of reducing sodium intake - encouraging behavioral changes that could be applied to other aspects of their lives - Goal: group average sodium intake of 80 mmol/24 h or less Assessment: - sodium excretion in 24-h urine samples. Potassium and creatinine excretion were also measured - sample validity: volume ⩾500 g (measured by weight) and collection time between 18 and 36 h - Blood pressure	Sodium excretion	3 years follow-up. From a 187 mmol/24 h baseline mean sodium excretion, net decreases were 44 mmol/24 h at 18 months and 38 mmol/24 h at 36 months in Sodium Reduction vs Usual Care. Corresponding net decreases in SBP/DBP were 2.0/1.4 mmHg at 18 months, and 1.7/0.9 mmHg at 36 months	mainly dietitians	an initial in person individual meeting, 10 weekly group meetings, four monthly group meetings and additional less frequent group sessions for the remainder of follow-up. Individual telephone and face-to-face contacts were offered as needed to facilitate maximum adherence	The same type and amount of counselling were offered to all participants in the active intervention: an initial in person individual meeting, 10 weekly group meetings, four monthly group meetings and additional less frequent group sessions for the remainder of follow-up. Individual telephone and face-to-face contacts were offered as needed to facilitate maximum adherence. The goal of the sodium reduction intervention was to achieve a group average sodium intake of 80 mmol/24 h or less by 6 months of follow-up, which was to be maintained to the end of the trial
2006	Cappuccio FP, Kerry SM, Micah FB, Plange-Rhule J, Eastwood JB. A community programme to reduce salt intake and blood pressure in Ghana [ISRCTN88789643]. BMC public health 2006;6:13	1,013 participants from 12 villages (628 women, 481 rural dwellers); mean age 55 years	community. Location: villages: in communal areas like churches, churchyards, schools, community centers	Intervention: - health education program: prevention of malaria, infective diarrheas and roundworm infection, diabetes, high blood pressure - In the intervention villages: additional advice was given not to add salt to food and in cooking, to limit the amount of koobi, momoni, kako and tilapia (salted fish), salted pigs' feet and salted beef and to soak the items in water overnight before eating them Tools: A standard health education package from the Ghana Ministry of Health was used in all the villages. Flip charts were the main medium of communication; they consisted of double-sided A3-sized sheets with a color picture on one side (shown to the participants) and written text on the other (facing the health visitor and used as a prompt) Frequency: meetings were held daily for the first week of the study and then once a week Duration: approximately one hour	Blood pressure	At six months the intervention group showed a reduction in systolic (2.54 mmHg [-1.45 to 6.54]) and diastolic (3.95 mmHg [0.78 to 7.11], p = 0.015) BP when compared to control. There was no significant change in UNa	Community health workers	training of CHW	reduction of salt intake
2008	Frisch S, Zittermann A, Berthold HK et al. A randomized controlled trial on the efficacy of carbohydrate-reduced or fat-reduced diets in patients attending a telemedically guided weight loss program. Cardiovasc Diabetol 2009; 8: 36	200 participants, age of 18 to 70 years and a BMI > 27 kg/m2	Heart Center North Rhine-Westphalia, Institute for Applied Telemedicine, Bad Oeynhausen, Germany	Common to all interventions Instructed all participants in an ambulatory training session and delivered diet books to all participants about their respective diets All participants were advised to reduce their daily energy intake by at least 500 kcal • Weekly, the actual body weight data had to be sent to using a mobile phone • The weight reduction program consisted of weekly nutrition education and dietary counselling by phone with a nutritionist during the first six months These calls were standardized according to weekly items about the respective diet, eating habits, and weight reduction, which allowed individual and problem-oriented advice. Each participant arranged her/his own appointment for weighting and calling. This regular weekly support was stopped during the second half-year	Weight loss	At study termination, weight loss was 5.8 kg (SD: 6.1 kg) in the low-carbohydrate group and 4.3 kg (SD: 5.1 kg) in the low-fat group (p = 0.065). In the low-carbohydrate group, triglyceride and HDL-cholesterol levels were lower at month 6 and waist circumference and systolic blood pressure were lower at month 12 compared with the low-fat group (P = 0.005–0.037)	Nutritionnist	Teleconselling. Both groups attended a weekly nutrition education program and dietary counselling by telephone, and had to transfer actual body weight data to our clinic weekly with added Bluetooth technology by mobile phone	Low carbohydrate diet: • developed by Ludwig et al. [9] and modified by Worm • The target macronutrient composition in the LOGI group was less than 40% of total energy intake from carbohydrates, more than 35% energy from fat, and 25% energy from protein Low fat-diet: • The target: more than 55% energy from carbohydrates, less than 30% energy from fat, and 15% energy from protein
2008	Shai I, Schwarzfuchs D, Henkin Y et al. Weight loss with a low-carbohydrate, Mediterranean, or low-fat diet. N Engl J Med 2008; 359: 229–241	322 participants, age of 40 to 65 years, BMI > 27, or type 2 diabetes or coronary heart disease, regardless of age and BMI	in a workplace at a research center with an on-site medical clinic	The participants were randomly assigned within strata of sex, age (below or above the median), BMI (below or above the median), history of coronary heart disease (yes or no), history of type 2 diabetes (yes or no), and current use of statins (none, < 1 year, or ≥ 1 year) with the use of Monte Carlo simulations. The participants received no financial compensation or gifts. The study was approved and monitored by the human subjects committee of Soroka Medical Center and Ben-Gurion University. Each participant provided written informed consent	Weight loss	The mean weight loss was 2.9 kg for the low-fat group, 4.4 kg for the Mediterranean-diet group, and 4.7 kg for the low-carbohydrate group (P < 0.001 for the interaction between diet group and time); among the 272 participants who completed the intervention, the mean weight losses were 3.3 kg, 4.6 kg, and 5.5 kg, respectively. The more favorable effects on lipids (with the low-carbohydrate diet) and on glycemic control (with the Mediterranean diet) suggest that personal preferences and metabolic considerations might inform individualized tailoring of dietary interventions	Registered dietician	The members of each of the three diet groups were assigned to subgroups of 17 to 19 participants, with six subgroups for each group. Each diet group was assigned a registered dietitian who led all six subgroups of that group. The dietitians met with their groups in weeks 1, 3, 5, and 7 and thereafter at 6-week intervals, for a total of 18 sessions of 90 min each. Six times during the 2-year intervention, another dietitian conducted 10-to-15-min motivational telephone calls + labelling	low-fat (AHA diet), restricted-calorie; Mediterranean, restricted-calorie; or low-carbohydrate (Atkins), non–restricted-calorie
2009	Keranen AM, Savolainen MJ, Reponen AH et al. (2009) The effect of eating behavior on weight loss and maintenance during a lifestyle intervention. Prev Med 49(1): 32–38	82 obese adults with BMI > 27 kg/m2	Oulu University Hospital, Finland	20 weeks. It included both individual and group counseling; after that ten visits every second week and the main idea was to help subjects to solve their problems related to diet and eating behavior. Counseling was conducted by a clinical nutritionist. The goal of dietary counseling was to improve eating frequency, to increase portion sizes of healthy foods and to reduce those of unhealthy foods, to favor a low fat diet high in unsaturated fats and low in saturated fats, to increase the intake of foods rich in fiber and calcium, and to decrease the consumption of sweets, alcohol and snack products. The goal of eating behavior counseling was to recognize and improve personal eating behavior. Homework was given to the subjects and they were encouraged to take the responsibility of the changes by themselves	Change of dietary habits	Eating behavior improved in both groups. Effect of counseling was -5.0 ± 5.7 kg compared with -2.4 ± 2.5 kg in the control group (p < 0.05 between the groups) during the first 6 months. At 18 months the weight loss results were -2.6 ± 6 kg and -0.7 ± 3.5 kg, respectively (NS)	Counseling was conducted by a clinical nutritionist	both individual and group counseling/the main idea was to help subjects to solve their problems related to diet and eating behavior	The goal of dietary counseling was to improve eating frequency, to increase portion sizes of healthy foods and to reduce those of unhealthy foods, to favor a low fat diet high in unsaturated fats and low in saturated fats, to increase the intake of foods rich in fiber and calcium, and to decrease the consumption of sweets, alcohol and snack products
2009	Morgan LM, Griffin BA, Millward DJ et al. Comparison of the effects of four commercially available weight-loss programmes on lipid-based cardiovascular risk factors. Public Health Nutr2009; 12: 799–807	300 overweight and obese men and women, aged 18 to 65 years who lived within 30 miles of a test centre and had a selfreported BMI between 27 and 40 kg/m2	five university centres across the UK, i.e. Surrey (Guildford), Nottingham, Ulster (Coleraine), Bristol and Edinburgh (Queen Margaret University College). Each centre aimed to recruit a cohort of sixty participants	Each participant undertook the diet to which they had been assigned for 6 months. For the group-based programmes (Weight Watchers and Rosemary Conley), participants arranged to attend the most geographically convenient class and the costs of joining and attending one class per week for 6 months were reimbursed on presentation of receipts. Both parent companies of Weight Watchers (www.weightwatchers.co. uk) and Rosemary Conley (www.rosemary-conley.co.uk) signed a contract committing to the provision of standard care. For Slim-Fast, the cost of up to two meal replacements per day was reimbursed on presentation of receipts, and a copy of the Slim-Fast Support Pack was provided. The Atkins group was given a copy of Dr Atkins’ New Diet Revolution(18). Control group subjects were asked to maintain their current diet and exercise pattern and were offered any of the diets for 6 months at the end of study, free of charge. All participants were able to claim reimbursement of travel costs. Diet group participants were instructed to follow the specific guidelines for each dietary programme, and every effort was made to avoid investigator management of, or interference in, participants’ food intakes. Participants attended the test centres on a 4-weekly basis, where they were weighed in light clothing and their blood pressure and waist circumference was measured. A fasting venous blood sample was taken from all participants at baseline, and after 8 and 24 weeks for the measurement of insulin, glucose and plasma lipids. Additional blood samples were taken monthly from the Atkins group to monitor renal function (urea, electrolytes and cystatin C)	Weight loss	efficacy of four popular weight-loss programmes on plasma lipids and lipoproteins as measures of CVD risk. Significant weight loss was achieved by all dieting groups (5–9 kg at 6 months) but no significant difference was observed between diets at 6 months. Significant weight loss was achieved by all dieting groups (5–9 kg at 6 months) but no significant difference was observed between diets at 6 months	Weihght watchers staff and Mary Conley staff	group sessions for Weight watchers and Mary conley (including PA), meal replacement for Slim Fast, Book for Dr Atkins diet	Weight-Watchers, Mary Conley, Slim-Fast, Atkins diet
2010	Huggins CE, Margerison C, Worsley A, Nowson CA. Influence of dietary modifications on the blood pressure response to antihypertensive medication. Br J Nutr. 2011;105(2):248–255	Ninety-four participants (38/56 females/males), aged 55·6 (SD 9·9) years, eligible if they were over 25 years of age and had a BP > / = 120 mmHg SBP or > / = 80 mmHg DBP at their second visit (mean of last three measurements) or home BP > / = 116 mmHg SBP or > / = 78 mmHg DBP. Ninety-four participants (38/56 females/males), aged 55·6 (SD 9·9) years	primary care	Control diet (CD), DASH diet (OD), LNAHK diet Dietary compliance assessed by 24 h-urinary sodium excretion and 24-h dietary record on the day before their visit with study staff **Dietary counseling** was overseen by the coordinating dietitian (C.M.) and provided by trained research staff Initial dietary counseling took between 10 and 30 min and was reinforced at every 2-wk contact. Participants were provided with printed materials explaining the diets in detail	Blood pressure	The LNAHK diet produced a greater fall in systolic BP (SBP) in those on antihypertensive therapy (-6·2 (SD 6·0) mmHg) than in those not on antihypertensive therapy (-2·8 (SD 4·0) mmHg) (P = 0·036), and this was greatest for those on renin-angiotensin system (RAS) blocker therapy (-9·5 (SD 6·4) mmHg) (interaction P = 0·007). The fall in SBP on the DASH-type diet, in those on therapy (overall -1·1 (SD 6·2) mmHg; renin-angiotensin blocker therapy -4·2 (SD 4·7) mmHg), was not as marked as that observed on the LNAHK diet	dietician + trained team	Dietary counseling by trained team + printed material	Control Diet (CD): low- potassium (and therefore low-magnesium), low-calcium diet DASH-type diet (OD): rich in vegetables, fruits and low-fat dairy products, with increased fish, nuts and legumes and a moderate sodium restriction. High in potassium (also magnesium and fiber), lower in saturated fat, and lower in sodium - 3 servings fish, 1 serving (100 g cooked) legumes, and 4 servings (30 g) unsalted nuts and seeds weekly, and limit red meat to 3 servings (100–120 g) weekly - Avoiding butter, no added table/cooking salt, and no salty foods - mono- or polyunsaturated margarine. Salt-reduced products (sodium 120 mg/100 g) and salt- reduced (50%) margarine Low-sodium, high-potassium diet (LNAHK). lower in sodium than DASH - no specific dietary recommendations for dairy products, fish, red meat, or the use of polyunsaturated and monoun- saturated fats - Salt-free bread and margarine provided, subjects advised to avoid added table/cooking salt and obviously salty foods - One additional serving of fruit/vegetables recommended (compared with OD) - recommendations: 4 servings fruit and 5 servings vegetables daily. servings legumes and 4 servings unsalted nuts and seeds weekly
2014	Esposito K, Maiorino MI, Petrizzo M et al.. The effects of a Mediterranean diet on the need for diabetes drugs and remission of newly diagnosed type 2 diabetes: follow-up of a randomized trial. Diabetes Care 2014;37:1824–30. 10.2337/dc13-2899	215 men and women with newly diagnosed type 2 diabetes		Participants in both groups were given detailed dietary advice by nutritionists and dietitians to achieve the dietary goals in monthly sessions in the first year and bimonthly sessions thereafter. Participants were also instructed how to record their intake using food models and actual weights or amounts in terms of common measures. Adherence to the diets was assessed by session attendance and review of the diet diaries. Participants in both groups were also advised to increase their level of physical activity, with programs tailored on the basis of the results of a baseline physical fitness test and safety concerns: gradual progression toward a goal of 175 min of moderate-intensity physical activity per week. All participants recorded occupational, household, and leisure time physical activity	Remission of type 2 diabetes	nutritionnists and dieticians	yes	no	The main goals of the dietary interventions were restriction of energy intake to 1,500 kcal/day for women and 1,800 kcal/day for men in both groups. The LCMD was rich in vegetables and whole grains and low in red meat, which was replaced with poultry and fish, with the goal of no more than 50% of calories from carbohydrates and no less than 30% calories from fat, with the main source of added fat 30–50 g of olive oil. The low-fat diet was rich in whole grains and restricted additional fats, sweets, and high-fat snacks, with the goal of no more than 30% of calories from fat and no more than 10% of calories from saturated fat
2015	Markota N, Rumboldt M, Rumboldt Z. Emphasized warning reduces salt intake: a randomized controlled trial.Journal of the American Society of Hypertension 2015;9:214–20	150 consecutive adult, treated hypertensives of either gender, registered in a family medicine practice in Mostar, Bosnia and Herzegovina	Hypertensive population—home-based	According to instructions in sealed envelopes: 1) the control group received individual information leaflets about the untoward effects of excessive salt consumption 2) the intervention group, in addition to the informational leaflets, received warning stickers to be mounted on all salt containers	Blood pressure	One month and 2 months later, a significant drop in BP, by 5.3/2.9 mm Hg, was observed in the intervention group as opposed to the control group (0.4/0.9 mm Hg). Decrease in Na24 positively correlated to BP lowering (r(2) = 0.5989; P < .0001)	?	written information vs written information + labelling home salt containers	Reducing salt intake
2013	Estruch R, Ros E, Salas-Salvadó J, Covas MI, Corella D, Arós F, Gómez-Gracia E, Ruiz-Gutiérrez V, Fiol M, Lapetra J, Lamuela-Raventos RM, Serra-Majem L, Pintó X, Basora J, Muñoz MA, Sorlí JV, Martínez JA, Fitó M, Gea A, Hernán MA, Martínez-González MA; PREDIMED Study Investigators. Primary Prevention of Cardiovascular Disease with a Mediterranean Diet Supplemented with Extra-Virgin Olive Oil or Nuts. N Engl J Med. 2018 Jun 21;378(25):e34. https://doi.org/10.1056/NEJMoa1800389. Epub 2018 Jun 13. PMID: 29,897,866	7447 subjects at high cardiovascular risk, but with no cardiovascular disease at enrollment	primary care,, retracted, 2018 updated version	Mediterranean diet—No total calories restriction was advised Dietitians ran individual and group dietary training sessions at the baseline visit and quarterly thereafter In each session, a 14-item dietary screener was used to assess adherence to the Mediterranean diet so that personalized advice could be provided to the study participants in these groups Intervention: Baseline and every 3 months: individual and group sessions (no more than 20 participants) LED BY Dieteticians - Assessment: 14 item dietary questionnaire (MD’s), 9 item questionnaire (low fat diet)– > The questionnaire responses were used to personalize the intervention for each participant, and to negotiate dietary changes - MD’s: supplementary food given (Extra virgin olive oil (1 L/week for the participant and their families) or mixed nuts (30 g/day: 15 g walnuts, 7.5 g hazelnuts, and 7.5 g almonds) MORE DETAILS: The PREDIMED dieticians are directly responsible for the dietary intervention. After two screening visits, participants randomized to each one of the three treatment arms had a face-to-face interview with the dietician and a group session (less than 20 subjects). A 14-point score of adherence to the MeDiet is a main tool to change dietary habits (Table 2).26–28 A similar 9-point score is used for the low-fat control group. For total fat intake, the recommendations given to participants in the low-fat diet group are opposite to those given to participants in the two MeDiet groups. The focus can be shifted from changing portion sizes, frequency of intake or cooking methods. We have reported an adequate effectiveness of the intervention after 1 year of follow-up.28 Because unsaturated fats like those contained in olive oil and nuts are still wrongly perceived as fattening, it has been particularly important to allay the fear of an eventual weight gain. Tactful exposition of recent scientific evidence,18,29–31 together with the fact that body weight did not change after 3 months of MeDiet intervention in the pilot phase of the PREDIMED study,26 have been instrumental in achieving this aim. The PREDIMED group sessions are organized separately for each of the three intervention groups. Participants are provided with written material (see: http://www.predimed.org and http://www.predimed.es) including descriptions of seasonal foods, shopping lists, weekly meal plans and cooking recipes. Olive oil and nut industry companies are committed to supplying for free the food supplements used in the study until December 2011. None of the investigators has any commercial interest with these food companies	Mortality rate	In the intention-to-treat analysis, there were 96 primary end-point events in the group assigned to a Mediterranean diet with extra-virgin olive oil (3.8%), 83 in the group assigned to a Mediterranean diet with nuts (3.4%), and 109 in the control group (4.4%). The respective incidence rates were 8.1, 8.0, and 11.2 per 1000 person-years, and the 5-year absolute risks were 3.6%, 4.0%, and 5.7%, respectively (Table 3). The unadjusted hazard ratios that used robust variance estimators to account for intracluster correlations were 0.70 (95% confidence interval [CI], 0.53 to 0.92) for a Mediterranean diet with extra-virgin olive oil and 0.70 (95% CI, 0.53 to 0.94) for a Mediterranean diet with nuts as compared with the control diet. In this study involving high-risk persons without cardiovascular disease, assignment to an energy-unrestricted Mediterranean diet supplemented with either extra-virgin olive oil or nuts was associated with a lower risk of major cardiovascular events over a period of 5 years than assignment to a control (low-fat) diet, with a relative difference of 30% and an absolute difference of 1.7 to 2.1 percentage points	For participants in the two Mediterranean-diet groups, dietitians ran individual and group dietary-training sessions at the baseline visit and quarterly thereafter. In each session, a 14-item dietary screener was used to assess adherence to the Mediterranean diet		three dietary intervention groups: a Mediterranean diet supplemented with extra-virgin olive oil, a Mediterranean diet supplemented with nuts, or a control diet. Traditional Mediterranean diet: rich in fruits and vegetables, but it also includes an abundance of legumes, a moderate intake of fish, dairy products, and wine, small portions of meat and poultry, and little consumption of candies (sweets). Participants in the two Mediterranean-diet groups received either extra-virgin olive oil (approximately 1 L per week) or 30 g of mixed nuts per day (15 g of walnuts, 7.5 g of hazelnuts, and 7.5 g of almonds) at no cost

The Dietary Approaches to Stop Hypertension (DASH) diet was the only diet proven to be effective for the three main CVD risk factors: blood pressure, lipid profiles and weight loss [18–21].

Twenty-six studies showed effective dietary interventions to reduce blood pressure. Eleven were individual dietary interventions, 10 were lifestyle interventions and five were community engagement studies. The interventions tested in these studies included a weight loss diet in ten studies and sodium reduction in the other sixteen studies. The interventions were led by dieticians in 14 studies, and by community health workers in one study. The provider was not described for 11 interventions. There were two or more providers of dietary interventions in 13 studies.

Seventeen studies presented effective dietary interventions for improving lipid profiles. Diets tested included the American Heart Association (AHA) diet [22, 23], the Hospital Contribution Fund of Australia diet (HCF) [22], the National Cholesterol Education Program (NCEP) recommendations [24], low-fat diets [22, 25–28], low-carbohydrate diets [27, 29], Mediterranean diet [17, 26, 30], commercial diets [31], and the low-calorie DASH diet [19]. Interventions were educational programs and mainly involved dieticians, instructors, psychologists, and nurses.

Fifty-five studies revealed effective diets for weight-loss. Ten studies focused exclusively on diet. The greatest weight loss was achieved with a low-carbohydrate diet but every diet involving calorie restriction was effective [19]. A Mediterranean, DASH diet, or low-fat diets should be promoted depending on individual CVD risk factors and patient preferences [23]. Forty-four studies involved combined interventions with diet plus physical activities. Mean weight loss was similar between dietary and lifestyle interventions (around 5 kg). Interventions were mainly led by dieticians and nutritionists and ranged from dietary advice to structured educational programs.

Two studies showed that physicians providing brief advice effectively reduced alcohol consumption [32, 33].

Eighteen community studies evaluated changes in purchases in favor of healthier food choices such as fat-free options. Outcomes were diverse and included cash register data, gallons of consumed milk, and sales increases. However, no cardiovascular health benefits could be extrapolated from these results.

Thirty-three studies evaluated recommendations to participants to follow a healthier diet using questionnaires. However, none of the questionnaires used were validated, meaning it was difficult to extrapolate cardiovascular health benefits from these results. Multiple professionals led the interventions including teachers, local stakeholders, physicians, nutritionists, dieticians, nurses, and computer systems.

The PREDIMED study was the only study aimed at improving cardiovascular outcomes that used a direct CVD prevention endpoint defined as major cardiovascular events. It implemented an energy-unrestricted Mediterranean diet supplemented with extra-virgin olive oil or nuts in a high CVD risk population [17]. After a 5-year follow-up, the end-point event incidence rates were 8.1 per 1000 person-years for the Mediterranean diet with extra-virgin olive oil, 8.0 per 1000 person-years for the Mediterranean diet with nuts and 11.2 per 1000 person-years for the control (low-fat) diet. Those on the PREDIMED diet had a lower risk of major cardiovascular events over a 5-year period than those on the control (low-fat) diet, with a relative difference of 30%. Another study, involving participants from the Arthritis, Diet, and Activity Promotion Trial (ADAPT), showed that the mortality rate of participants randomized to an 18-month weight loss intervention was lower than controls (hazard ratio = 0.5, 95% confidence interval 0.3–1.0) seven years after the intervention [16].

Lifestyle interventions associated a dietary intervention with at least one other intervention and effectively reduced CVD morbidity. Eighty-five studies combined diet with between one and three different interventions including physical activity, lifestyle management, smoking cessation, alcohol reduction, and involved healthcare professionals. Twelve other studies involved communities and changes to workplace and public layouts. Sixty studies combined just diet and physical activity making this the most common combination. This was more effective on blood pressure [17], lipids [17], weight loss and body composition [17]. The effects of these lifestyle interventions were more sustained than diet alone [17]. Any physical activity type was effective if it was supervised by the research team. Professionals involved were diverse and included physicians, dieticians, nurses, and lay people.

The Finnish Diabetes Prevention study [34] and the Diabetes Prevention Program (DPP) [35] demonstrated that a structured lifestyle program including weight loss effectively reduced diabetes incidence. Six other studies included in the matrix studied the implementation of these programs. They were effective in multiple settings [36, 37] and had long-term efficacy [38]. Delivery of such programs using conference calls [39]or community health workers [40] was effective.

## Discussion

To our knowledge, this is the first systematic review of international clinical guidelines following the ADAPTE process to identify effective dietary interventions for CVD prevention in primary care or community settings. A matrix of 383 effective dietary interventions to prevent CVD was created. The SPICES literature review was created to provide a matrix of effective, detailed interventions on diet, physical activity, and smoking regulation for CVD primary prevention to implement into vulnerable communities. For this purpose, the DASH diet appears to be the most effective in preventing CVD as it concurrently reduces blood pressure, improves the lipid profile, and leads to weight loss. This is a variation of the Mediterranean diet and provides indications about the amounts of each nutrient that should be consumed. It can be adapted to the cultural habits of every community and different CVD patient profiles. Combining diet with other interventions such as physical activity and smoking cessation increases efficacy. Physicians providing brief advice is shown to be an efficient strategy to address excessive alcohol consumption. Overall, guidelines did not provide detailed strategies to implement the recommendations in communities.

Guidelines had contradictory recommendations on supplements. They were all rated on their editorial independence following the AGREE II tool. External review of their content was performed before publishing. However, their conclusions were contradictory despite being based on the same body of evidence. External review should admonish institutional bias. However, external review within the same profession can lead to opinions that are distanced from the evidence.

The different dietary interventions were led by a variety of professionals including physicians, dieticians, health workers, nurses, and lay people. This is encouraging as it reveals that diverse professionals can successfully implement and lead these interventions, which is important for areas with poor access to healthcare professionals. Furthermore, training lay people to lead dietary interventions for CVD prevention could be a possible solution for public health authorities, particularly in low-income countries.

This review underlines the lack of recommendations and studies adapted to the low- and middle-income countries. In fact, only five studies in the final matrix were conducted in these countries [21, 41–44]. This meant stakeholders and researchers had to estimate the transferability of high-income country prevention strategies, especially for dietary interventions. However, the similar spread of junk food in these countries has already been demonstrated [45].

The matrix produced from this review enables any researcher or caregiver to choose a validated intervention to implement in primary care. However, some intervention implementation details, implied psychological models and intervention durations, are missing from the matrix as they were not described in the studies or were too old. This affects the reproducibility of these effective interventions, and the imprecisions could lead to failure. Furthermore, implementing the current, very general recommendations may miss the precise key required for interventions to be successful.

The selected studies mainly focused on individual changes rather than community approaches (126 studies versus 38 studies). The individual benefit of dietary interventions for primary CVD prevention was small in some studies, for example losing a few kilograms in weight or improving blood pressure by several millimeters of mercury. This therefore raises the question as to whether it is ethical to ask individuals to commit such efforts. Furthermore, during clinical trials, individuals find it easier to stick to a dietary intervention but in real-life situations, they are often unlikely to make long-term, significant dietary changes. Cheaper, more plentiful, higher-fat foods make following strict diets harder and also contribute to the increasing prevalence of CVD risk factors such as obesity, high blood pressure and increased lipid levels making primary CVD prevention an important public health issue [46]. Since the included studies presented interventions for individuals, it was difficult to extrapolate the results to the general population. However, some individual interventions such as salt restriction could be implemented in the general population for example by regulating salted food items. This could reduce salt intake and subsequently reduce blood pressure therefore reducing CVD mortality [47].

### Strengths and limitations

One study strength was using international clinical guidelines as information sources with searches being conducted on specific guideline databases, TRIP and G-I-N. However, these databases were incomplete, meaning other databases and a purposive search were required to complete the selection process and find the missing guidelines. The selection process was rigorous due to the two double-blind phases and the involvement of a scientific committee. Information bias was controlled through evaluation triangulation. It was assumed that selection and publication biases were previously addressed by each guideline provider. Assessing each guideline with the AGREE II tool meant that the researchers expected to select guidelines with the most reliable bibliographies and intervention implementation information could be captured. Some authors suggest selecting guidelines just by scoring the rigor and development domain [48]. However, this misses the stakeholder involvement and applicability domains which are other areas of concern for implementation. This review had no specific strategy to assess risk of individual study biases. It was assumed that the guideline developers dealt with this. However, it should be limited by retaining only high-quality guidelines which is one of the reasons for using the ADAPTE process.

Searching for implementation information resulted in a deeper search into the guidelines. This produced an underestimated workload that ADAPTE was supposed to prevent. An evaluation cascade occurred, firstly of guidelines, then recommendations and finally cited studies. The diverse recommendation rating systems and the fact that links between recommendations and the bodies of evidence were sometimes missing meant selecting recommendations with a high level of evidence was impossible. Mean study publication dates were unexpectedly out of date. Guidelines should cite the most up to date studies and this review shows that this is not the case. It is unclear whether this is because guidelines do not regularly update their body of evidence or whether there is little recent research on dietary interventions. No common measure was found to present study efficacy since population, outcomes, and statistical processes were disparate. Even for highly recommended interventions, only trends can be estimated. Furthermore, recommendations are based on a body of evidence that is largely based on surrogate endpoints.

## Conclusion

On an individual basis, this review indicates that implementing the DASH diet combined with any kind of physical activity, smoking reduction or cessation, and brief advice about reducing alcohol consumption effectively reduces CVD risk factors. However, this seems difficult to expand to the wider population without government support to implement regulations such as reducing salt content in processed food.

## Data Availability

All data generated or analyzed during this study are included in this published article and its supplementary information files.

## Abbreviations

AHA: American Heart Association
ADAPT: Arthritis, Diet, and Activity Promotion Trial
AGREE II: Appraisal of Guidelines Research and Evaluation II
CVD: Cardiovascular diseases
DASH: Dietary Approaches to Stop Hypertension
ESC: European Society of Cardiology
HCF: Hospital Contribution Fund of Australia diet
ICCC: Innovative Care for Chronic Conditions
NCEP: National Cholesterol Education Program
PIPOH: Population, Intervention, Professional/Patient, Outcome, Healthcare setting
SSA: Sub-Saharan Africa
SIGN: Scottish Intercollegiate Guideline Network
TRIP: Turning Research Into Practice
WHO: World Health Organization
